# Nuclear Translocation of Glutaminase GLS2 in Human Cancer Cells Associates with Proliferation Arrest and Differentiation

**DOI:** 10.1038/s41598-020-58264-4

**Published:** 2020-02-10

**Authors:** Amada R. López de la Oliva, José A. Campos-Sandoval, María C. Gómez-García, Carolina Cardona, Mercedes Martín-Rufián, Fernando J. Sialana, Laura Castilla, Narkhyun Bae, Carolina Lobo, Ana Peñalver, Marina García-Frutos, David Carro, Victoria Enrique, José C. Paz, Raghavendra G. Mirmira, Antonia Gutiérrez, Francisco J. Alonso, Juan A. Segura, José M. Matés, Gert Lubec, Javier Márquez

**Affiliations:** 1 Departamento de Biología Molecular y Bioquímica, Canceromics Lab, Facultad de Ciencias, Universidad de Málaga, 29071 Málaga, Spain and Instituto de Investigación Biomédica de Málaga (IBIMA), Málaga, Spain; 2 Proteomics Lab, Central Facility Core, University of Málaga, 29071 Málaga, Spain; 3 Private Medical University of Salzburg, Salzburg, A5020 Austria; 4 Institute of Science and Technology Austria, Am Campus 1, A-3400 Klosterneuburg, Austria; 5 Department of Pediatrics, Indiana University School of Medicine, Indianapolis, IN 46202 USA; 6 Departamento de Biología Celular, Genética y Fisiología, Facultad de Ciencias, Universidad de Málaga, Instituto de Investigación Biomédica de Málaga (IBIMA). Centro de Investigación Biomédica en Red sobre Enfermedades Neurodegenerativas (CIBERNED), 29071 Málaga, Spain

**Keywords:** Cancer metabolism, Proteomics

## Abstract

Glutaminase (GA) catalyzes the first step in mitochondrial glutaminolysis playing a key role in cancer metabolic reprogramming. Humans express two types of GA isoforms: GLS and GLS2. GLS isozymes have been consistently related to cell proliferation, but the role of GLS2 in cancer remains poorly understood. GLS2 is repressed in many tumor cells and a better understanding of its function in tumorigenesis may further the development of new therapeutic approaches. We analyzed GLS2 expression in HCC, GBM and neuroblastoma cells, as well as in monkey COS-7 cells. We studied GLS2 expression after induction of differentiation with phorbol ester (PMA) and transduction with the full-length cDNA of GLS2. In parallel, we investigated cell cycle progression and levels of p53, p21 and c-Myc proteins. Using the baculovirus system, human GLS2 protein was overexpressed, purified and analyzed for posttranslational modifications employing a proteomics LC-MS/MS platform. We have demonstrated a dual targeting of GLS2 in human cancer cells. Immunocytochemistry and subcellular fractionation gave consistent results demonstrating nuclear and mitochondrial locations, with the latter being predominant. Nuclear targeting was confirmed in cancer cells overexpressing c-Myc- and GFP-tagged GLS2 proteins. We assessed the subnuclear location finding a widespread distribution of GLS2 in the nucleoplasm without clear overlapping with specific nuclear substructures. GLS2 expression and nuclear accrual notably increased by treatment of SH-SY5Y cells with PMA and it correlated with cell cycle arrest at G2/M, upregulation of tumor suppressor p53 and p21 protein. A similar response was obtained by overexpression of GLS2 in T98G glioma cells, including downregulation of oncogene c-Myc. Furthermore, human GLS2 was identified as being hypusinated by MS analysis, a posttranslational modification which may be relevant for its nuclear targeting and/or function. Our studies provide evidence for a tumor suppressor role of GLS2 in certain types of cancer. The data imply that GLS2 can be regarded as a highly mobile and multilocalizing protein translocated to both mitochondria and nuclei. Upregulation of GLS2 in cancer cells induced an antiproliferative response with cell cycle arrest at the G2/M phase.

## Introduction

Altered metabolism is a hallmark of cancer^1^ and the term “metabolic reprogramming” has been coined to describe the whole range of metabolic abnormalities accompanying tumorigenesis and metastasis^2^. Increased glutamine (Gln) uptake and glutaminolysis are key metabolic traits that have been consistently found in a wide range of human and experimental cancers^3^. The term “Gln addiction” is now widely used to reflect the strong dependence shown by most cancer cells for this essential nitrogen substrate after metabolic reprogramming^4^. Glutaminase (GA, EC 3.5.1.2) proteins control the first step in the glutaminolytic process: the conversion of Gln to glutamate (Glu) and ammonium ions^5^. In mammals, four different isoenzymes have been characterized so far:^6^ the alternative spliced KGA and GAC proteins, encoded by the *Gls* gene^7,8^, and the LGA and GAB isoforms coded by the second GA gene, *Gls2*^9^. The GAB isozyme is the product of the canonical full-length transcript coded by the *Gls2* gene^10^, while the short LGA transcript appears by alternative transcription initiation and uses an alternative promoter^11^.

It is well documented that many tumors show increased GA activity which is positively correlated with their malignancy^3^. GA and glutaminolysis play key roles in tumorigenesis which are not only related to energy generation, but also with the supply of nitrogen and carbon skeletons for macromolecule biosynthesis^12^. We initially reported that inhibition by antisense technology of *Gls* expression (KGA isoform) allowed the reversion of Ehrlich ascites tumor cells to a more differentiated and less malignant phenotype^13^. Recent works are starting to uncover the differential expression of GA isoenzymes in cancer, along with their regulation by oncogenes and tumor suppressor genes. Thus, it has been shown that oncogene c-Myc derepresses *GLS* expression in several cancer cell types through a miRNA mechanism^14^. GLS isoforms are also upregulated by certain oncogenic signaling pathways, such as the small Rho GTPases^15^, which activate the GLS isoform GAC through a mechanism dependent on nuclear factor-kappa B (NF-κB)^16^. Hence, the link between GLS isoforms and neoplastic transformation seems supported by convincing evidence in human gliomas, lung and liver tumors.

While GLS upregulation correlates with proliferating stages and malignancy in many types of cancer and experimental tumors, little is known about the role of GLS2 in tumorigenesis. We first postulated a completely different role for GLS and GLS2 isoforms in cancer based on their relative expression patterns in human leukemia, breast cancer cells, and hepatocellular transformation^17^. The process of malignant transformation shifts the pattern of GA expression in such a way that GLS becomes upregulated while GLS2 is frequently repressed; for instance, transformed liver cells, like HepG2, return to a fetal-like phenotype, characterized by a high rate of cell proliferation and prevalence of GLS isoforms over GLS2 ones, which predominate in normal nonproliferating hepatocytes^17^. Co-expression of GLS and GLS2 transcripts has been reported in established cancer cell lines of colon, hepatoma, leukemia and breast, although protein data suggest that GLS isoforms would account for the majority of GA activity in these human tumor cells^17,18^. In fact, GLS2 expression is repressed in highly malignant glioblastoma (GBM)^19^, as well as in human liver and colon cancers^20–22^. Furthermore, GLS2 was confirmed as a target gene of the tumor suppressor p53, in such a way that p53-controlled enhanced GLS2 expression was linked to a tumor-suppressive response, including reduced growth and colony formation of tumor cells^20,21^. Importantly, a recent study implicated GLS2 in miRNA regulation through Dicer stabilization, upregulation of miR-34a and repression of Snail and metastasis in hepatocellular carcinoma (HCC) cells^23^. Therefore, a strikingly different pattern, opposed to GLS, is becoming evident for GLS2 in many malignancies, but not all^3^. In this work, we extended our previous finding of a nuclear expression for GLS2 in brain cells^24,25^ by showing that GLS2 is located in both mitochondria and nuclei of human cancer cells. GLS2 overexpression elicits an antiproliferative response involving mitochondria and cell nucleus; the nuclear targeting correlated with a p53-dependent tumor suppressive mechanism allowing proliferation arrest of human cancer cells at G2/M. Thus, overexpression or derepression of *GLS2* appears as a potential novel therapeutic strategy for certain types of cancer.

## Results

### Intracellular distribution of GLS2 in human tumor cells

**Figure 1:**
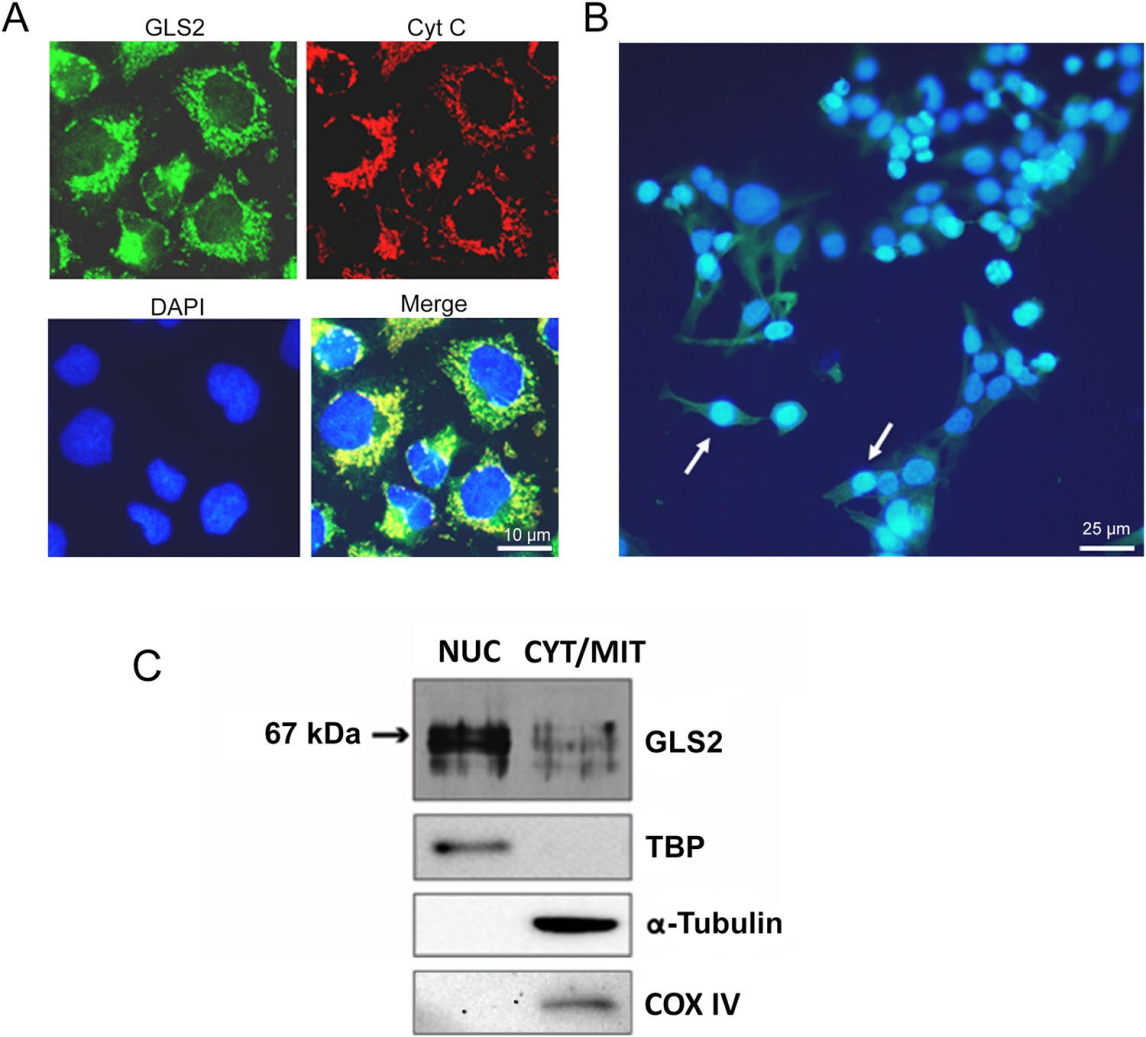
Intracellular localization of GLS2 in human HCC cells. Immunocytochemistry of HepG2 cells using affinity-purified isoform-specific anti-GLS2 antibodies revealed a major cytosolic punctate immunostaining for GLS2 and a minor nuclear immunostaining (**A1, GLS2**). The mitochondrial GLS2 mark was further confirmed by double immunofluorescence studies, using cytochrome c as a mitochondrial marker: essentially most of the extranuclear cytosolic GLS2 staining overlapped with the mitochondrial marker (**A2, Cyt C**). Cell nuclei were stained with DAPI (**A3, DAPI**) and the co-localization of GLS2-cyt c and GLS2-DAPI was also revealed using a triple overlay (**A4, Merge**). (**B**) The minor nuclear mark was also confirmed using double immunofluorescence labeling with anti-GLS2 antibodies (green) and cell nuclei stained with DAPI (blue): only a discrete number of HepG2 cells showed GLS2-reactive nuclei (19.3%, n = 352 individual cells). (**C**) Confirmation of the nuclear GLS2 mark by subcellular fractionation: nuclear and cytosolic fractions isolated from HepG2 cells were probed with anti-GLS2 antibodies by immunoblot analysis (bands cropped from the original Western blot shown in Supplemental Fig. S1). The molecular mass of the standard BSA protein marker is indicated on the left. Antibodies against TATA-binding protein (TBP), α-tubulin and COX IV were used as organelle markers for nucleus, cytoplasm and mitochondria, respectively. NUC, nuclear fractions; CYT/MIT, cytosolic (mitochondrial) fractions.

Although a nucleocytoplasmic location for GLS2 protein was reported in mammalian brain cells^24,25^, the subcellular localization of GLS2 in human cancer cells has not been yet elucidated. Immunocytochemistry studies in HepG2 human cancer cells using affinity-purified isoform-specific anti-GLS2 antibodies revealed a cytosolic punctate immunostaining for GLS2, strongly suggestive of mitochondrial localization, along with a minor nuclear immunostaining only found in a discrete cell population (Fig. 1-A1). Similar results were obtained with T98G and SH-SY5Y human cancer cells (results not shown). Accordingly, the minor nuclear mark was also confirmed using double fluorescence labeling with anti-GLS2 antibodies and cell nuclei stained with DAPI: only a discrete number of HepG2 cells (19.3%) showed GLS2-reactive nuclei (Fig. 1-A3/A4,1B). The mitochondrial mark was further confirmed by double immunofluorescence studies, using cytochrome c as a mitochondrial marker: essentially most of the extranuclear cytosolic GLS2 staining overlapped with the mitochondrial marker (Fig. 1-A2/A4).

Mitochondria is the default and basal location of GA isoenzymes in mammalian tissues and cells under physiological conditions^5,6^, although previous immunofluorescence microscopy and biochemical data demonstrated nuclear localization of GLS2 in neurons and astrocytes^24,25^. To confirm the presence of GLS2 protein in the nucleus of human cancer cell lines, HepG2 cells were fractionated into nuclear and cytosolic pools. Then, they were probed by Western blot using isoform-specific anti-GLS2 antibodies: the presence of GLS2 proteins in both fractions was detected (Fig. 1C). On the other hand, no GLS isoforms were detected by Western blot in the assayed nuclear fractions (results not shown), which were devoid of any significant cytoplasmic or mitochondrial contaminations as revealed by probing the blots with anti-α-tubulin and anti-cytochrome c oxidase (complex IV) specific antibodies (Fig. 1C).

### Nuclear localization of GLS2 in human cancer cells transduced with GLS2-tagged constructs

**Figure 2:**
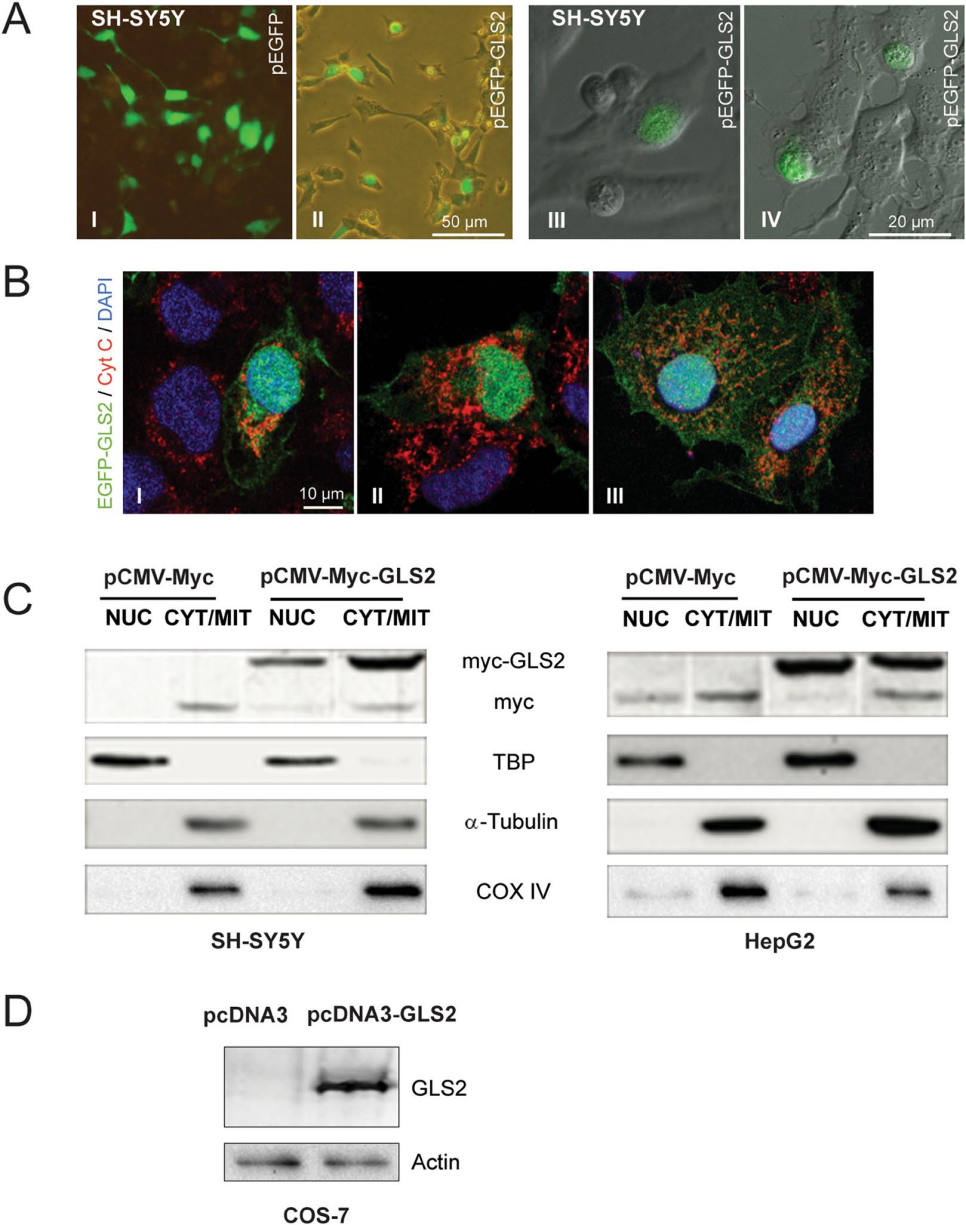
Nuclear location of GLS2-tagged proteins ectopically expressed in human cancer and COS-7 cells. The full-length ORF of GLS2 was cloned in the pCMV-Myc and pEGFP expression vectors. (**A**) SH-SY5Y cells were transfected with pEGFP-GLS2 and GLS2-tagged proteins visualized by fluorescence (**A-I, A-II**) and confocal laser scanning microscopy (**A-III, A-IV**). The construct pEGFP-24, expressing the GFP protein alone, was employed as transfection control for these experiments (**A**–**I**). Approximately, 40% of transfected cells showed nuclear GLS2 (n = 200). Transfections of transformed African green monkey kidney fibroblast (COS-7) cells were done with two expression vectors: pEGFP-GLS2 (**B**) and pcDNA3-GLS2 (**D**) which yield the full-length GLS2 isoform EGFP-tagged and untagged, respectively. EGFP-tagged GLS2 proteins were visualized by confocal microscopy after immunostaining with anti-cyt c antibodies to label mitochondria and DAPI staining for cell nuclei (**B**). SH-SY5Y and HepG2 cells were transfected with the plasmids p-CMV-Myc and p-CMV-Myc-GLS2 (**C**). Then, nuclear (NUC) and cytoplasmic/mitochondrial (CYT/MIT) fractions were isolated and Myc-tagged GLS2 proteins detected by Western blots probed with anti-c-Myc tag antibodies (bands cropped and grouped from the original Western blots shown in Supplemental Fig. S1). The construct pCMV-Myc was employed as transfection control for Western blotting experiments. Antibodies against TATA-binding protein (TBP), α-tubulin and COX IV were used as organelle markers for nucleus, cytoplasm and mitochondria, respectively. (**D**) *left lane*, wild-type COS-7 cells do not express any appreciable GLS2 proteins; *right lane*, a clear protein band was detected after transfection with pcDNA3-GLS2 construct (full-length original Western blot shown in Fig. S1). Both lanes were also revealed with antibodies against β-actin as loading control.

To unambiguously demonstrate nuclear targeting of GLS2 proteins in human cancer cells, we decided to use orthogonal methods not relying on the recognition of proteins by anti-GLS2 antibodies. Expression constructs were prepared with the whole ORF of GLS2 fused to a c-Myc epitope tag or to EGFP fluorescent protein. After transfection in SH-SY5Y and HepG2 cells, ectopically expressed GLS2-tagged proteins were visualized either by fluorescence and confocal microscopy or by Western blots of nuclear extracts probed with anti-c-Myc antibodies (Fig. 2). The overexpressed GFP protein control labeled all the cell body (Fig. 2A-I), because GFP can travel in and out of the nucleus by passive diffusion due to its small size^26^. Neuroblastoma SH-SY5Y cells expressing GFP-tagged GLS2 showed a fluorescent pattern labeling the cell body, as expected from proteins which are co-expressed in mitochondria and nuclei, although some cells showed a predominant nuclear fluorescence (Fig. 2A-II). Even more, a few individual cells displayed only the nuclear GLS2 mark without appreciable cytosolic labeling (Fig. 2A-III/IV). Many SH-SY5Y cells were not transfected with the EGFP-GAB plasmid, a result that would be anticipated taking into account their known resistance to cellular transfection (ThermoFisher Scientific technical note, https://www.thermofisher.com/). The nuclear and cytosolic (mitochondrial) localizations of GLS2 were also revealed in cancer cells transfected with the c-Myc-tagged GLS2 constructs: Western blots of transfected SH-SY5Y and HepG2 cells showed a clear band in both compartments at the expected molecular mass of taggeg-GLS2 (Fig. 2C). The protein band corresponding to the endogenous c-Myc protein was also detected in most lanes probed with the c-Myc specific antibody (Fig. 2C).

Additionally, we asked whether GLS2 would be also targeted to the nucleus in a cell type with null expression of this isoform. To answer this question, we chose COS-7 cells that do not have appreciable GLS2 protein expression, but display a good level of expression after transfection with a pcDNA3-GLS2 vector (Fig. 2-D). Then, COS-7 cells were transfected with a pEGFP-GLS2 plasmid and the fluorescent fusion protein visualized by confocal microscopy. Ectopic expression of GFP-GLS2 in COS-7 labeled the cell body showing an abundant punctate cytosolic mark, along with a clear nuclear fluorescent staining (50,7% of transfected cells showed a clear nuclear mark, n = 182 individual cells) (Fig. 2B). Some DAPI-stained nuclei (dark blue) appear green and greenish-blue due to the intense GFP-tagged GLS2 mark (Fig. 2B-I and II). On the other hand, cytoplasmic GLS2 label partially overlapped with the mitochondrial marker (cyt c), giving rise to an orange-yellowish stain (Fig. 2B-I and III), although part of the cytosolic GFP-tagged GLS2 proteins did not merge with cyt c (Fig. 2B-I and III). Image J analysis of co-localizations yielded Pearson correlation values of pixel intensities of 0.29 and 0.35 for the EGFP-GLS2/DAPI and EGFP-GLS2/cyt c pairs, respectively (Fig. S2).

Therefore, we conclude that GLS2 protein can be shuttled to the nucleus after ectopic expression in human cancer cells; even more, GLS2 protein expressed in cell types with null GLS2 expression (COS-7) was also able to be targeted to the cell nuclei.

### Differentiation agents induce translocation of GLS2 into cancer cells nuclei

**Figure 3:**
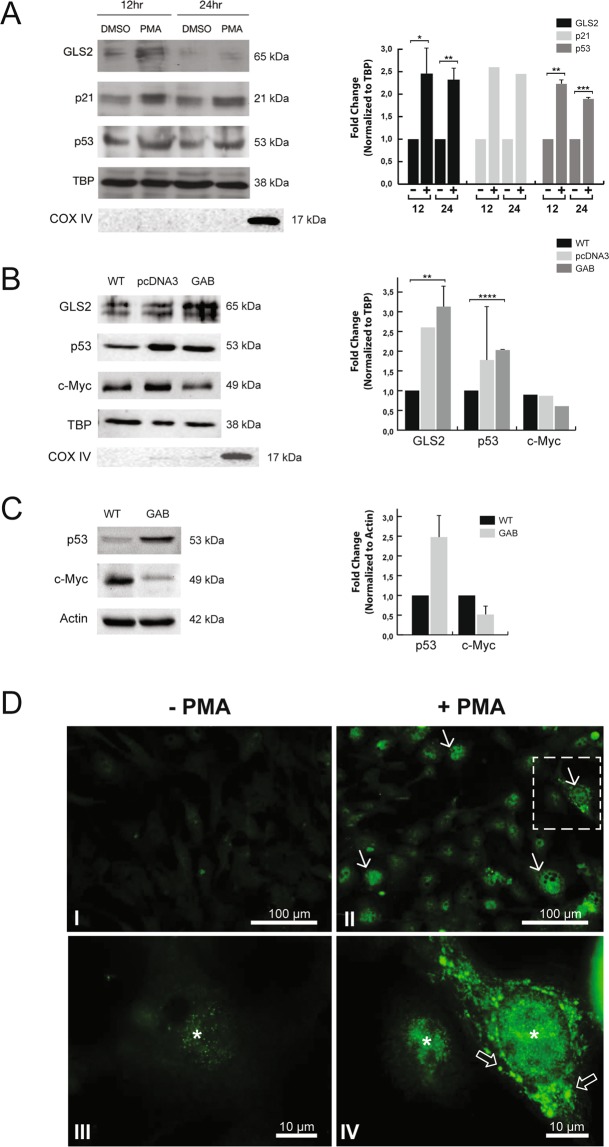
Differentiation agent PMA increases mitochondrial and nuclear GLS2 protein levels in SH-SY5Y and HepG2 cancer cells. (**A**) Representative Western blot of nuclear fractions isolated from SH-SY5Y cells after incubation with 1 µM PMA for 12 h and 24 h or dimethyl sulfoxide (DMSO). Blots were probed with antibodies against GLS2, p21, p53 and TATA-binding protein (TBP) used as a loading control. Antibodies against COX IV were used to assess cytoplasmic (mitochondrial) contamination. (**B**) Representative Western blot of nuclear fractions isolated from human GBM T98G cells: wild-type (WT), stably-transfected with vector alone (pcDNA3) and stably-transfected with GLS2 (GAB). Blots were probed with antibodies against GLS2, p53, c-Myc and the loading control TBP. Antibodies against COX IV were used to assess cytoplasmic (mitochondrial) contamination. (**C**) Representative Western blot of whole cellular extracts isolated from human GBM T98G cells: wild-type (WT) and stably-transfected with GLS2 (GAB). Blots were probed with antibodies against p53 and c-Myc using β-actin as loading control. (**D**) Confocal microscopy images of GLS2 immunofluorescence in HepG2 cells treated with PMA. Cells grown on cover slips were treated with vehicle DMSO (I–III) or 1 µM PMA in DMSO (II–IV) for 6 h. After incubation, cells were stained with rabbit antibody to GLS2 and Alexa 488 conjugated secondary antibody. Induction of differentiation (II–IV) clearly increased the nuclear (asterisk) and cytoplasmic accumulation of GLS2 protein (arrows) versus untreated cells (I-III). GLS2 also showed some minor nuclear localization without treatment (asterisk in III). Open arrows in IV indicate an intense perinuclear immunostaining of GLS2. Scale bars: 100 μm (I,II) and 10 μm (III, IV). Original Western blots of Fig. 3-A, B and C are shown in Supplemental Fig. S1. Densitometric analysis of the protein bands, normalized to the loading control, are shown on the right of panels A, B and C. For all panels, the values represent the mean (n ≥ 2) and the error bars represent ± SD, except for p21 in A and c-Myc in B (n = 1). Paired Student’s t test was done between PMA treated and untreated cells, and T98G-GAB vs T98G-WT or T98G-pcDNA3.*p < 0.05, **p < 0.01, ***p < 0.005, ****p < 0.001.

SH-SY5Y and HepG2 cells treated with the differentiation agent phorbol 12-myristate 13-acetate (PMA) showed an increased nuclear accrual of GLS2 above the basal level observed in untreated cells (Figs. 3-A and 3-D, respectively). This enhanced expression was seen at early incubation times for neuroblastoma cells (12–24 h, Fig. 3-A) and HepG2 cells (6–24 h, Fig. 3-D). The increase of GLS2 expression after PMA stimulation was detected in both mitochondria and nucleus; thus, the Pearson correlation coefficient values for co-localization, obtained after quantification of cytoplasmic and nuclear immunofluorescence signals of GLS2, cyt c and DAPI staining in individual HepG2 cells (n > 75 per condition), significantly increased in PMA-treated cells: from 0.69 to 0.77 for GLS2-DAPI (p < 0.0001) and from 0.65 to 0.71 for GLS2-cyt c (p < 0.0001) (Fig. S3). Indeed, the number of cells with nuclear GLS2 showed a fourfold rise in the presence of PMA, raising from 19.3% under control conditions (n = 352 cells) to 85.5% in cells exposed to PMA (n = 159 cells).

Furthermore, SH-SY5Y cells forced to differentiate with PMA also showed increased protein levels of active p53 and its related partner cyclin-dependent kinase inhibitor p21 (Fig. 3-A): the upregulation of p53 and p21 positively correlated with that of GLS2 in cell nuclei of SH-SY5Y cells treated with PMA for 12 h and 24 h (Fig. 3-A). This result associates nuclear translocation of GLS2 with stimulus-inducing differentiation of cancer cells, reinforcing the view of GLS2 upregulation as a hallmark of quiescent differentiated cell states, but in sharp contrast to the opposite oncogenic role played by GLS isoenzymes^17^. In addition to cancer cells treated with PMA, another model with increased nuclear levels of GLS2 is the GBM cell line T98G-GAB, stably transfected with the full-length ORF of GLS2, which possesses a less-malignant phenotype compared with the wild-type T98G parental cell line^27^ (Fig. S4). The nuclear extracts of these more differentiated and slow-growing GBM cells showed enhanced levels of GLS2 (Fig. 3-B), in agreement with the results previously seen in PMA-treated cancer cells. We further assessed the expression levels of the tumor suppressor gene p53 and oncogene c-Myc in T98G-GAB cells versus their wild-type T98G counterparts. The immunoblot analysis showed an enhanced p53 expression in both nuclear fractions (Fig. 3-B) and whole cellular extracts (Fig. 3-C), while the oncogenic protein c-Myc was also down-regulated but only in cellular extracts (Fig. 3-C).

### Subnuclear location of GLS2 in tumor cells

**Figure 4:**
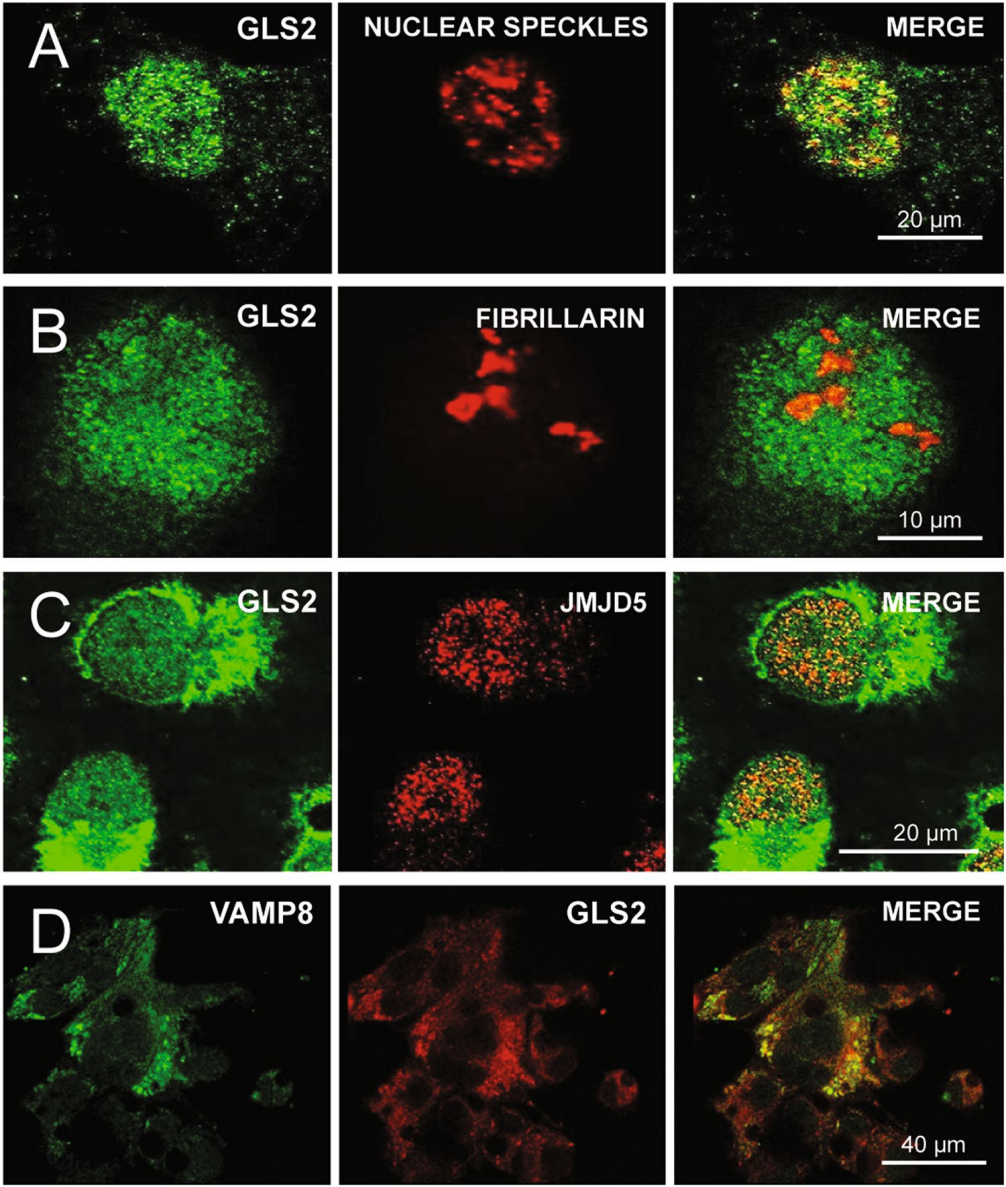
Subnuclear localization of GLS2. Confocal microscopy of HepG2 cells treated with PMA. (**A**) GLS2-Nuclear speckles. Double immunofluorescence labeling with anti-GLS2 antibodies (*left*, green) and anti-nuclear speckles antibodies (*center*, red); *right*, view of double labeled cells showing only a few overlapping spots between the two immunolabels. (**B**) GLS2-Nucleolus. Double immunofluorescence labeling with anti-GLS2 antibodies (*left*, green) and antibodies against fibrillarin specific for cell nucleoli (*center*, red); *right*, merge view of double labeled cells: the nucleolar marker did not overlap with the nuclear GLS2 mark. (**C**) GLS2-Nucleoplasm. Confocal microscopy of HepG2 cells treated with PMA after double fluorescence labeling with anti-GLS2 antibodies (*left*, green) and anti-JMJD5 histone demethylase antibodies, a marker specific for cell nucleoplasm (*center*, red). (**D**) Double immunofluorescence labeling and confocal microscopy of HepG2 cell employing anti-VAMP8 (green) and anti-GLS2 (red) antibodies: the merge panel shows a high degree of overlapping marks.

To get a deep insight into the nuclear function of GLS2 we next addressed its subnuclear localization: adscription of the GLS2 mark to a specific subnuclear organelle may give us a clue of its potential nuclear function(s). We used two models with an enhanced nuclear targeting of GLS2: HepG2 cells stimulated with PMA and T98G-GAB cells overexpressing GLS2. We performed double immunofluorescence labeling and confocal microscopy with affinity-purified isoform-specific anti-GLS2 antibodies, along with commercial antibodies specific for two subnuclear organelles: nuclear speckles (Fig. 4-A) and nucleoli (Fig. 4-B). The choice of these particular organelles was because the nuclear labeling pattern revealed for GLS2 sometimes showed a condensed appearance resembling the topography of these organelles. The confocal microscopy results indicated that GLS2 was not exclusively associated with nuclear speckles (although some overlapping was detected, Fig. 4-A) or cell nucleolus (Fig. 4-B), but was instead spread around the nucleoplasm. To confirm this widespread mark in the nucleoplasm, we also used a third nuclear antibody raised against histone H1 demethylase as a marker of the nucleoplasm (Fig. 4-C). The GLS2 mark was distributed along the nucleoplasm, mimicking the nuclear pattern observed for the histone H1 demethylase protein (Fig. 4-C), without clear segregation or overlapping with known subnuclear structures (Figs. 4-A and 4-B). Quantification of co-localization data for GLS2 in subnuclear compartments by analysis of cell nuclei yielded very low Pearson correlation coefficients (Fig. S5), confirming a widespread nucleoplasmic location of GLS2 without significant co-localization with speckles or nucleolus.

On the other hand, in PMA-treated HepG2 cells a strong perinuclear immunostaining was clearly visible (Fig. 3-D). This may suggest that nuclear import of GLS2 could be mediated by a previous mobilization of mitochondria around the cell nucleus and hence being imported into the nucleus. Alternatively, the perinuclear ring detected in PMA-stimulated HepG2 cells could arise due to a mobilization of vesicles carrying their protein cargo toward the nucleus. To assess this latter alternative, we next performed double immunolabeling combining anti-vesicle-associated membrane protein 8 (VAMP 8) antibodies, chosen as a marker of traffic vesicles, with anti-GLS2 antibodies (Fig. 4-D). The confocal microscopy results showed an intense perinuclear region where VAMP 8 and GLS2 immunostainings clearly overlapped in PMA-treated HepG2 cells (Fig. 4-D). The perinuclear space appears crowded with vesicles, in coincidence with the perinuclear mark detected for GLS2 protein; hence, vesicle-mediated nuclear import of GLS2 in PMA-treated cells cannot be discarded.

### Increases in the nuclear and mitochondrial accrual of GLS2 protein associate with G2/M cell cycle stage

**Figure 5:**
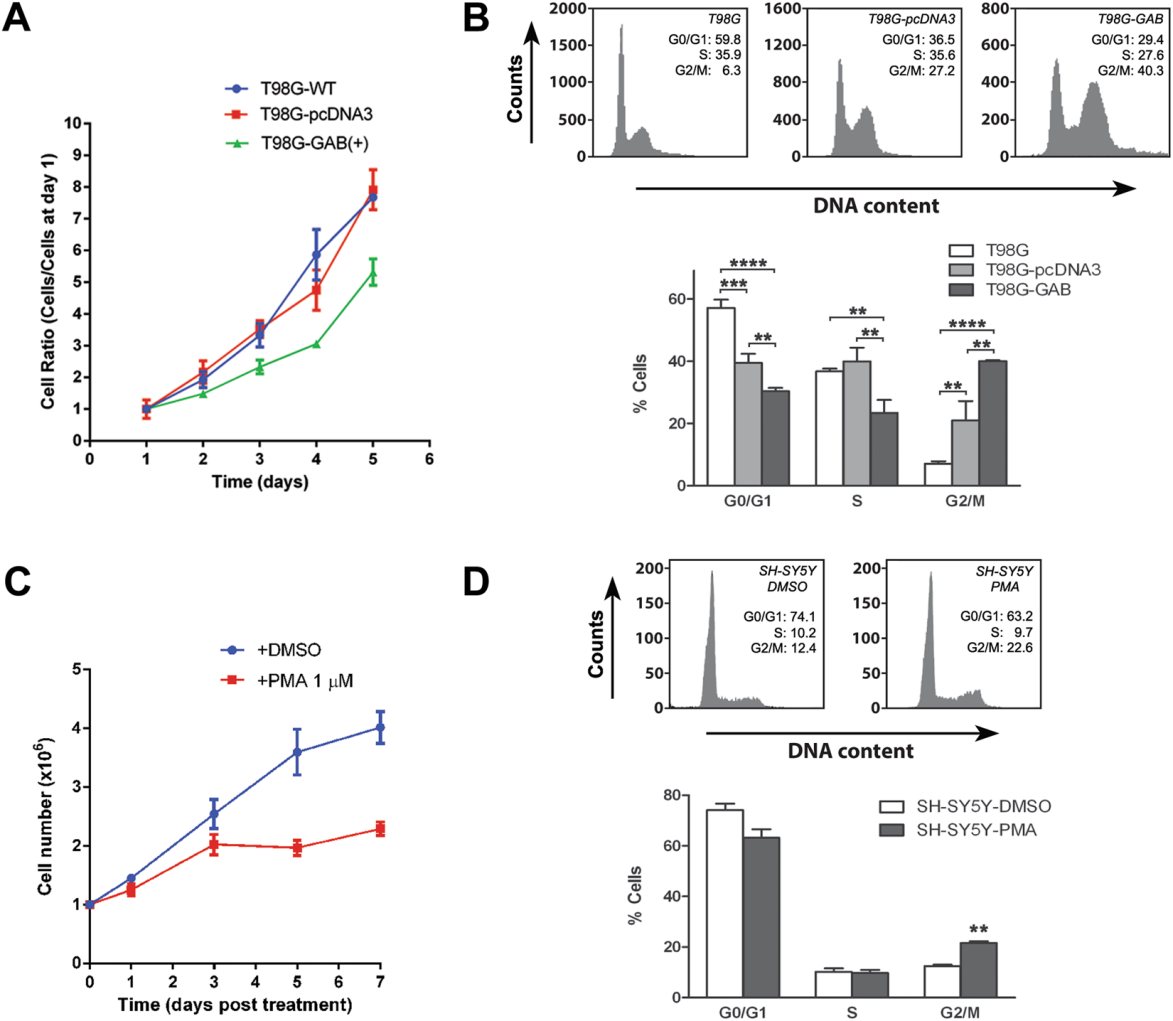
Cell cycle studies in human GBM T98G-GAB cells and human neuroblastoma SH-SY5Y cells treated with PMA for 24 h. (**A**) For assessing the effect of GAB overexpression on cell proliferation, 8 × 10^4^ T98G, sham-transfected T98G-pcDNA3 and GAB overexpressing T98G-GAB(+) cells were seeded per well in 6-well plates. Cells were detached and counted in triplicate (independent wells) at days 1, 2, 3, 4 and 5 after seeding. Cell number was normalized to the value at day 1 for each cell line variant assayed. (**B**) FACS sorting of T98G, T98G-pcDNA3 and T98G-GAB cells after 24 hours in culture. The chart indicates the percentage of total cells found at each cell cycle stage. Wild-type T98G cells and cell lines stably transfected with the full-length ORF of GLS2 (T98G-GAB) and vector pcDNA3 alone (T98G-pcDNA3) were analyzed. (**C**) For assessing the effect of PMA on cell proliferation rate, 1 million SH-SY5Y cells were seeded per well in eight 6-well plates. After 24 hours, once cells were just attached to the surface, cell culture medium was supplemented with 1 µM PMA dissolved in DMSO (experimental group) or 0.033% DMSO for the control group (same DMSO concentration than the experimental group). Cells were detached and counted in quadruplicate (independent wells) at days 1, 3, 5 and 7 after treatment. (**D**) FACS sorting of SH-SY5Y control cells (DMSO) and SH-SY5Y cells treated with PMA after 24 hours in culture. The chart indicates the percentage of total cells found at each cell cycle stage. Three distinct samples were analyzed for each independent human cancer cell line assayed. Panel B (ANOVA): **p < 0.01; ***p < 0.001; ****p < 0.0001. Panel D (Student’s t test): **p < 0.01.

Next, we aimed to ascertain whether tumor cells overexpressing GLS2 constitutively (T98G-GAB) or after induction of differentiation by PMA treatment (SH-SY5Y) shift their cellular populations toward cell cycle stages compatible with lower proliferation. First, we determine the growth curves for glioma cell lines T98G-WT, T98G-pcDNA3 (sham-transfected) and T98G-GAB. Glioma cells overexpressing GAB decrease their proliferation (Fig. 5A) vs wild-type and sham-transfected controls, a result previously reported by our group^27^. For example, mean doubling times were 1,824 ± 0,034 for GAB-transfected cells vs 1,188 ± 0,039 for the WT counterparts (Fig. S4A). Then, we performed a cell cycle analysis to detect shifts in the distribution of cancer cells after GLS2 overexpression. A clear trend was revealed in T98G-GAB cells: a strong increase in the population of cells in the G2/M phase, along with a decrease in S and G0/G1 stages, was detected as compared with WT and sham-transfected cell counterparts (Fig. 5B). For SH-SY5Y neuroblastoma cells, differentiation agent PMA also induced a clear inhibition of cell proliferation with cell numbers half of those shown by DMSO-treated control cells (Fig. 5C). Moreover, DNA flow cytometric analysis of neuroblastoma SH-SY5Y cells indicated that treatment with PMA also resulted in a G2/M arrest, when compared with control untreated cells (Fig. 5D). Thus, two cancer cell models with increased GLS2 expression and nuclear accrual showed a stabilization of the cell cycle in the G2/M phase, in sharp contrast with GLS isoforms whose expression was upregulated in G1 and S phases in association with cell proliferation^28^.

### Posttranslational modification of human GLS2 protein

**Figure 6:**
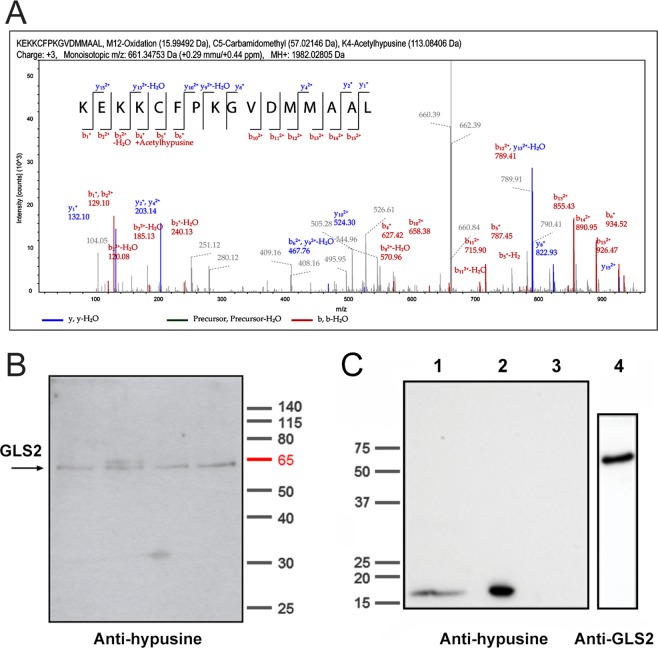
Posttranslational modification of recombinant human GLS2 protein with acetylhypusine. (**A**) Representative spectrum of a GLS2 peptide with acetylhypusine modification. The peptide KEKKCFPKGVDMMAAL (from amino acid 329 to 344 of the GLS2 sequence) appears as a charge + 3 peptide with a monoisotopic m/z of 661.34753 Da (+0.29 mmu/+0.44 ppm). MH: 1982.02805 Da, including C-5 carbamidomethyl (57.02 Da), M-12 oxidation (15.99 Da) and K4-Acetylhypusine (113.08 Da). (**B**) Validation of GLS2 hypusination by immunoblot analysis using specific rabbit polyclonal anti-hypusine antibodies. Four different fractions containing affinity-purified recombinant human GLS2 from two different GIP-affinity purifications were analyzed. The anti-hypusine antibodies recognized a band in all four lanes corresponding to the known molecular mass of mature GLS2 protein (29). Electrophoresis was done in 12% polyacrylamide Bis-Tris gel with PageRuler Prestained Protein Ladders (Thermo Scientific) (positions of markers indicated at the right end). (**C**) Positive and negative controls for hypusination. A whole protein extract from mouse brain and rat prefrontal cortex (20 µg each) were employed as positive controls by revealing a band of the hypusinated eIF5A protein at approx. 17 kDa (lanes 1 and 2). Purified human recombinant GLS2_56–602_ protein expressed in bacteria was chosen as a negative control. It was analyzed in 10% SDS-PAGE gel and revealed with anti-hypusine antibodies (lane 3) or anti-GLS2 antibodies (lane 4). Positions of molecular mass markers are shown at the left end of panel C. Full-length gels and blots are shown in both panels B and C.

Finally, we set up a baculovirus expression system and affinity purification to obtain functional human GLS2 for analysis of posttranslational modifications^29^. The human GLS2 protein was purified by affinity chromatography employing the Glutaminase-Interacting Protein (GIP) as a specific ligand (Fig. S7). The purified GLS2 fractions were digested with trypsin and chymotrypsin and then submitted to LC-MS/MS analysis. Higher energy collision dissociation (HCD) allowed better sequence coverage than collision-induced dissociation (CID) with both proteolytic enzymes, ranging from 57.6% to 77.1%, while the number of unique GLS2 peptides identified was slightly higher for the chymotrypsin digestion (Suppl. Tables 1 and 2). Overall, the consolidated sequence coverage from both trypsin and chymotrypsin analysis was 89% (Fig. S8), demonstrating the identity of the GLS2 protein analyzed. With regard to posttranslational modifications, LC-MS/MS analysis identified the chymotryptic peptide –KEKKCFPKGVDMMAAL- being modified with acetylhypusine in the fourth lysine residue (K-336 of the whole GLS2 amino acid sequence) (Fig. 6-A). Hypusine (N-ε-(4-amino-2-hydroxybutyl) is an unusual amino acid formed at specific lysine residues after a two-step enzymatic process^30^. Because hypusination is a rare modification, we looked for additional confirmation of this posttranslational modification (PTM) found in the GLS2 amino acid sequence. To validate the LC-MS data, we used Western blotting employing specific anti-hypusine polyclonal antibodies raised against a synthetic hypusine-containing peptide^31^. Affinity-purified human GLS2 was probed by immunoblot analysis: the anti-hypusine antibody recognized a single protein species at the molecular mass (~63 kDa) corresponding to the mature GLS2 protein, in four distinct fractions from two independent purifications (Fig. 6-B). A doublet was also seen in one fraction, corresponding to the non-processed precursor form of human GLS2 which co-purify in some extent with the mature protein (Fig. S7)^29^. As a positive control, a whole mouse brain and rat prefrontal cortex extracts were probed with the anti-hypusine antibody: a protein band of approximately 17 kDa was clearly revealed (Fig. 6-C, lanes 1 and 2), corresponding to the known molecular mass of eukaryotic translation initiation factor 5A (eIF5A), the only known protein containing the amino acid hypusine^30^. As negative controls, we included human recombinant GLS2_56–602_ protein expressed in bacteria (Fig. 6-C, lanes 3 and 4), a relevant GLS2 control because hypusination does not occur in bacterial expression systems, as well as purified recombinant Glutaminase-Interacting Protein (GIP) and the human KGA isoform expressed in baculovirus (Fig. S6).

## Discussion

Emerging experimental evidences support that GA isozymes exhibit multiple functions^32^. Elucidation of the respective role of each GA isoform may be of profound physiological significance not only in normal conditions, but also in pathological states like cancer where GLS and GLS2 isoenzymes seem to play apparently opposing roles^3^. This fact has been clearly demonstrated in recent studies, where inhibition of cancer proliferation and reversion of the malignant phenotype were achieved by knocking-down GLS or by upregulation of GLS2 expression^33,34^. While overwhelming evidence points to GLS isozymes as oncogenic proteins, underlying metabolic reprogramming leading to cancer growth and proliferation in Gln-addicted tumors, the role of GLS2 isozymes in tumor biology has not yet been elucidated and needs further clarification^35^. In the current study, we demonstrated that GLS2 can be targeted to the nucleus of cancer cells. The nuclear localization of GLS2 may give us new insights to understand non-glutaminolysis functions of this isoenzyme in cancer.

We made use of three distinct experimental approaches for elucidating the nuclear localization of GLS2 proteins in human tumor cells. Our first approach, using immunocytochemistry with isoenzyme-specific antibodies, revealed co-localization of GLS2 in both mitochondria and nuclei, although mitochondrial expression was always much higher than in cell nuclei under basal conditions. The second one was based on subcellular fractionation and immunoblot analysis with the same GA antibodies: nuclear and mitochondrial locations of GLS2 were consistently found. The third approach involved an orthogonal method of validation that does not rely on anti-GLS2 antibodies: expression of GLS2-tagged proteins in cancer cells. Two different tags, EGFP and c-Myc epitope, were employed; in both cases, a fraction of the GLS2-tagged protein was located in cell nuclei, as detected by immunofluorescence or by Western blot with anti-c-Myc specific antibodies. On the other hand, the GA band presented in nuclear fractions isolated from human cancer cell lines cannot be ascribed to GLS isoforms by two main reasons: first, we always use affinity-purified isoenzyme-specific antibodies for cell immunostaining and Western blots, which only recognize GLS or GLS2 isoforms, but not both of them^24,25^ (Figs. S9 and S10); second, neither KGA nor mitochondrial contamination, that might give rise to a GLS band, were present in nuclear extracts employed in the analysis. Apart from human cancer cells, we also demonstrated a nuclear location of GLS2 in COS-7 cells ectopically expressing the human protein. Consistent with this finding, we previously reported that human GLS2 protein expressed in Sf9 cells transduced with recombinant baculovirus also localized in both mitochondria and nucleus^29^.

It has been shown that GLS2 expression is downregulated in certain types of cancer like gliomas^19^, colon^22^ and HCC^20–23^. Accordingly, the nuclear concentration of GLS2 in HepG2, T98G and SH-SY5Y cancer cells, under basal conditions, is very low compared with the mitochondrial pool of this isoform. Therefore, we next tried to search for conditions which may enhance the nuclear accumulation of GLS2 and to ascertain whether or not this increased nuclear translocation is associated with the reversion of the malignant phenotype and inhibition of cell proliferation. Induction of differentiation of HepG2 and SH-SY5Y cells with PMA increases the nuclear translocation of GLS2 in both cell types at early incubation times. Moreover, upregulation of GLS2 was also evident in mitochondria; hence, the tumor suppressive effects elicited by GLS2 overexpression might involve mitochondrial and nuclear contributions. Of note, GL2 mRNA levels significantly increased in parallel with the terminal differentiation of SH-SY5Y cells treated with retinoic acid (RA)^36^. These authors demonstrated enhanced neurite outgrowth after ectopic GLS2 expression in the absence of RA, along with a 40% increase of intracellular ATP levels; therefore, they concluded that GLS2 participates in the regulation of neuronal differentiation under the transcriptional control of TAp73, a p53 family member^36^. Further evidences linking GLS2 with cell differentiation were recently obtained after the discovery of its regulation by TAp63, another transcription factor belonging to the p53 family^37^. Specifically, GLS2 and TAp63 expression increased during differentiation of primary human keratinocytes, whilst depletion of GLS2 inhibited skin differentiation^37^. Very recently, a nuclear location of GLS isoforms was firstly reported in the prostate cancer cell line PC-3, associated with a decreased transcriptional activity of peroxisome proliferator-activated receptor γ (PPARγ)^38^. This role of GLS in transcriptional regulation occurs through a direct interaction with PPARγ and independent of its catalytic activity, which further reinforces the view of GA as multifaceted moonlighting proteins^39^.

Our current work further suggests a causative link between GLS2 expression and tumor suppression. Transfection of human GBM cells with GLS2 induces a marked change in the cell’s transcriptome^27^, leading to a more differentiated and less-malignant phenotype characterized by a strong inhibition of cell proliferation^27,40^ (Fig. S4). The different phenotypes of these *in vitro* models were also revealed by studying their growth in adult bovine serum, thus avoiding non-physiological levels of cysteine in standard cell culture media which have been recently reported to induce an increased reliance on glutamine anaplerosis^41^. Wild-type and sham-transfected T98G GBM cells grow and proliferate under such conditions; however, T98G-GAB cells showed a barred proliferation (Fig. S4-B). Of interest, our results indicate that induction of GLS2 expression in GBM cells also affects the regulation of tumor suppressor genes and oncogenes. Notably, active p53 was upregulated in T98G-GAB cell nuclei, whilst c-Myc, known to be negatively regulated by p53^42^, was significantly downregulated in T98G-GAB whole cell extracts. Induction of p53 was also noted in the GBM cell line transfected with the vector alone, whereas p21 was barely detected in the GBM cell lines analyzed. A similar result was obtained in PMA-treated SH-SY5Y cells, where GLS2 overexpression was paralleled by marked increases of active p53 and the anti-proliferative cyclin-dependent kinase inhibitor p21 in cell nuclei. Thus, increased nuclear translocation of GLS2 associates with inhibition of proliferation and reversion of the transformed phenotype. This tumor suppressor role elicited by GLS2 induction would be mediated, at least in part, by p53-dependent mechanisms. Consistent with these results, the p53-dependent GLS2 upregulation and its tumor suppressive role in HCC was associated with mitochondrial activation and enhanced antioxidant defense^20,21^ and, more recently, with inhibition of Rac1 activation and metastasis suppression^38^. Notably, the activity of glutathione reductase and levels of GSH were significantly higher in T98G-GAB cells than in GLS2-negative T98G wild-type cells^43^. In this context, the unique kinetic properties of GLS2 may represent an important advantage by allowing generation of GSH even in a Glu-enriched intracellular milieu, because GLS2 is barely inhibited by the reaction product Glu^29^, in sharp contrast with GLS isoforms^5,6^.

In relation to the nuclear translocation of GLS2, a perinuclear GLS2-positive staining was clearly revealed in HepG2 cells forced to differentiate with PMA. It is well known that the perinuclear region surrounding the nucleus may be transitorily enriched in mitochondria, for example to provide energy-rich compound needed to satisfy the high-energy requirements of nuclear processes, like nucleocytoplasmic transport of proteins^44^. Therefore, the mitochondrial GLS2 would be translocated to the cell nucleus from this perinuclear mitochondrial ring. However, double immunofluorescence labeling and confocal microscopy studies, using anti-VAMP8 (a vesicle marker) and anti-GLS2 antibodies, suggest that nuclear transport would be vesicle-mediated, because the perinuclear zone was highly enriched with VAMP8-containing transport vesicles which strongly overlap with the GLS2 staining. In this context, it is noteworthy that VAMP8 was the most upregulated transcript in glioma T98G-GAB cells stably transfected with GLS2^27^, while reduced VAMP8 levels have been associated to invasive phenotype in breast cancer cells^45^.

We studied the subnuclear localization of GLS2 in an attempt to get a deep insight into its nuclear role(s). Three subnuclear compartments were assessed: nuclear speckles, nucleolus and the nucleoplasm. Although the shape of GLS2 immunostaining sometimes appears as condensed granules with a punctate aspect, neither nuclear speckles nor nucleoli immunolabeling marks showed a significant overlap with the GLS2 staining, as assessed by confocal microscopy analysis and quantitative study of co-localization with Image J. Instead, the GLS2 mark was diffusively distributed throughout the nucleoplasm, showing remarkable similarity with the pattern revealed for the Jumanji JMJD5 histone demethylase/hydrolase. Therefore, nuclear GLS2 does not seem to be involved in functions typically ascribed to speckles and nucleoli, mainly pre-mRNA splicing and pre-rRNA and pre-tRNA processing, respectively. However, the cell nucleus is an extremely dynamic organelle; particularly, nuclear proteins and RNAs are constantly in exchange from their particular nuclear compartments with the nucleoplasm^46^. Thus, despite the fact that GLS2 showed a wide distribution in the nucleoplasm, we cannot rule out that it may be transitorily associated with some nuclear compartments under specific conditions (cell cycle stage, cell type, the presence of stressors, etc.).

An increased nuclear accrual of GLS2 occurred in parallel with the inhibition of proliferation of human cancer cells. The antiproliferative response was induced either by differentiation agents (PMA in SH-SY5Y and HepG2 cells) or by overexpression of GLS2 (T98G-GAB cells). Interestingly, FACS analysis of cell populations consistently detected a stop of the cell cycle at the G2/M stage coincident with the increased nuclear translocation of GLS2. In addition, cell cycle arrest at G2/M was paralleled by upregulations of active p53 and cyclin-dependent kinase inhibitor p21, whereas oncogene c-Myc was simultaneously downregulated. It is well known that p21 induction occurs in cells that undergo p53-dependent cell cycle arrest or apoptosis, because the p53-p21 axis is a central pathway for cell cycle control and tumor suppression^47^. Thus, it is tempting to speculate that nuclear GLS2 participates in the growth-arrest program preventing unlimited cell proliferation, which may explain why certain tumors (e.g. gliomas, hepatomas and lung cancers) showed the opposite behavior: silencing of GLS2 and upregulation of GLS isoforms to keep active their proliferative program^3^. Consistent with these findings, overexpression of GLS2 in human lung, liver and colon cancer cells induced significant reductions in growth and proliferation^21,22^. In HCC, this effect was greatly mediated by negative regulation of the PI3K/AKT pathway^48^. Furthermore, ectopic expression of GLS2 in liver SMMC-7721 and colon HCT116 cancer cells also induced a G2/M arrest^22^, in accordance with the results here described for human GBM and neuroblastoma cells. This G2/M arrest is consistent with the observed accumulation of GLS2, p53 and p21 protein levels, in agreement with the functional tumor suppressor role postulated for GLS2 as novel target of the p53 family of tumor suppressors^20,21,43^. Despite these evidences, GLS2 also suppressed the malignant phenotype of human GBM of different tumorigenic potentials and genetic backgrounds, including p53(─) cells^27,40^. Nevertheless, additional data characterizing the nuclear roles of GLS2 are still needed to definitively substantiate a direct link between cell cycle arrest and nuclear translocation of GLS2.

Cell proliferation is an energetically demanding process and, thereby, tightly coupled with metabolic control^28^. Glutamine is a key nutrient for cell cycle progression and glutaminolysis is required for the G1 to S transition along with glycolysis; however, unlike glucose, Gln is the only substrate essential for the progression through S phase into cell division, as revealed by the high activity and protein levels of GLS isozymes, which do not decline until the G2/M phase is reached^49^. In sharp contrast, the nuclear accrual of GLS2 in neuroblastoma and glioma cells occurs in parallel with cell cycle arrest at the G2/M stage. Interestingly, a completely different response is found for cancer cells grown in the absence of Gln or after silencing/inhibition of GLS isoform: cells enter the S phase but fail to progress into G2/M^49,50^. Therefore, Gln deprivation and GLS inhibition arrest cancer cells in S phase, while overexpression of GLS2 and exposure to differentiation agents elicit stop at G2/M phase. Thus, the anti-proliferative response induced by blocking GLS or by stress due to nutrient (Gln) removal seems to occur by different mechanisms to those triggered by GLS2 overexpression. Importantly, GLS2 show anti-oncogenic roles independently of its glutaminolysis function in HCC, allowing metastasis suppression through Rac1 activation^43^ and repression of the epithelial-mesenchymal transition through the Dicer-miR-34a-Snail axis^23^.

In chemotherapy, efforts to increase G2/M arrest of cancer cells have also been associated with enhanced apoptosis. Interestingly, the generation of reactive oxygen species (ROS) by treatment with oxidizing agents, like arsenic trioxide or hydrogen peroxide, synergizes with GLS2 overexpression to suppress malignant properties of T98G glioma cells^33,40^. Of note, GLS2 evoked in these cells lower c-Myc and Bcl-2 protein levels, as well as a higher expression of the pro-apoptotic Bid protein, which may explain the marked fall in cell number mediated by GLS2^33,40^ (Fig. S4). Thus, glioma cell lines overexpressing GLS2 undergo significant apoptosis after the G2/M arrest, a response also found in epithelial and hematological malignancies treated with selective chemotherapeutic agents that did not affect normal cell counterparts^51^. In this context, it is noteworthy that stable transfection with GLS2 inhibited cell growth of T98G GBM through downregulation of O6-methylguanine-DNA methyltransferase (MGMT) and, thus, sensitized cells to alkylating agents as temozolomide and carmustine^40,52^. Consequently, GLS2 upregulation may facilitate chemotherapeutic intervention in addition to inhibiting glioma growth. Alternatively, other strategies targeting GLS isoforms have been successful in cancer cells where glutaminolysis becomes essential, as in mutant isocitrate dehydrogenase 1/2 (IDH1/2) GBM subtypes^53^, or in combined therapies devised to counteract the glutaminolysis-based drug resistance response, as reported in T-cell acute lymphoblastic leukemia patients treated with anti-Notch1 compounds^54^. However, no GLS inhibitor has been proved successful in the clinic so far. Notably, if GLS2 has location-specific functions independent of glutaminolysis in cell nuclei, it could become a promising target. Actually, the pattern of GLS and GLS2 expression differentially modulate the clinical outcome of cancers^55^ and is becoming a useful metabolic signature to diagnose responders to GLS cancer therapy^56,57^.

GLS2 isoform is a protein that shuttles in and out of the nucleus without having a classical nuclear export signal or nuclear localization signal required for transportation via exportin/importin pathways^24^. Therefore, we looked for specific posttranslational modifications on the GLS2 protein as a putative selection mechanism for nuclear import and/or function. Hypusination of human recombinant GAB was identified by mass spectrometry and validated by immunoblot analysis. In eukaryotic cells, this rare posttranslational modification has been only found in the eukaryotic translation initiation factor 5A (eIF5A), which plays a key role in cell proliferation and survival^30^. The hypusine residue is essential for interaction of eIF5A with specific nucleotide sequences of mRNAs during translation and also for its nucleocytoplasmic shuttling^58^. The hypusine-modified lysine in the GAB primary structure (K-336) is located in an unfolded segment of the protein, a short turn of 9 residues connecting two alpha helices in a sequence highly enriched in polar amino acids (KKCFPKGVD), which strongly suggest that this hypusinated lysine is exposed to the external medium. Interestingly, the exposed hypusine of eIF5A becomes essential for interaction with exportin Xpo4 allowing its nucleocytoplasmic shuttling^59^. Thus, the exposed hypusine residue might be a structural determinant needed for nuclear import of GLS2 through nuclear transport receptors or for specific interaction with nucleotides required in a putative transcriptional regulatory role, although further experiments will be needed to assess the functional relevance of this PTM in the GLS2 protein expressed in human cancer cells, as well as the role of the hypusination enzyme machinery in cancer growth and proliferation.

## Conclusion

We demonstrated that GLS2 can be targeted to the cell nucleus in human GBM, neuroblastoma and HCC cells. Our studies demonstrated a correlation between nuclear targeting and the antiproliferative response induced by GLS2 in human cancer cell lines, but further studies are needed to elucidate whether nuclear GLS2 is causatively linked to this response. The results strongly suggest that GLS2 upregulation has tumor suppression activity and could help to rewire cellular metabolism toward a normal non-proliferative phenotype, providing a new strategy to combat some types of cancer where GLS2 is frequently silenced. Furthermore, expression levels of GLS2 might also potentially be used as a prognostic and/or diagnostic factor in some human malignancies.

## Methods

### Cell cultures

Human hepatoblastoma HepG2 cells (European Collection of Cell Cultures, Cambridge, UK) were cultured as described previously^17^. The SH-SY5Y human neuroblastoma cell line (European Collection of Cell Cultures, Cambridge, UK) were grown in RPMI (Sigma) medium supplemented with 10% FCS, penicillin (100 U/mL) and streptomycin (100 µg/mL). The T98G and T98G-GAB human glioma cell lines were cultured as described previously^27^. COS-7 cells were cultured in Dulbecco’s Modified Eagles Medium (DMEM) (BioWhittaker) supplemented with 10% FCS (v/v) and same antibiotics mentioned above. Cultures were incubated in a humidified atmosphere at 37 °C with 5% CO_2_. All cell lines were checked to be free of mycoplasma contamination.  The three GBM cell lines (T98G wild-type (T98G-WT), T98G-pcDNA3 sham-transfected and T98G-GAB overexpressing GAB isoenzyme) were also grown in adult bovine serum alone, to assess the effects of culturing cells in serum-levels of cystine. Cells (5 × 10^5^) were cultured in 6-well plates following the conditions described by Muir *et al*.^41^. After one week of culture, cells were counted using a hemocytometer and trypan blue, to account only for viable cells.

### Expression constructs and cellular transfections

*Eco*RI and *Xho*I endonuclease restriction sites flanking the coding region of the human GLS2 cDNA were constructed by PCR using the 2408-bp GLS2 cDNA in pGEM-T-Easy as a template^60^. After double digestion and purification, the insert was cloned into the EcoRI/XhoI site of pcDNA3 (Invitrogen). COS-7 cells were transfected with the pcDNA3-GLS2 construct using the co-precipitation method with calcium phosphate^61^. Expression of human GLS2 in transduced COS-7 cells was analyzed after 48 h. The pEGFP-GLS2 and pCMV-Myc-GLS2 vectors were constructed as follows. The human GLS2 cDNA was amplified and flanked by the required endonuclease restriction sites by PCR. After digestion and purification, the inserts were cloned into the pEGFP-C1 (XhoI/BamHI) and pCMV-Myc-N (XhoI) vectors (Clontech). The orientation and sequence of the GLS2 inserts in all the constructs were confirmed by sequencing.

For transfection with pEGFP-GLS2 and pCMV-Myc-GLS2, SH-SY5Y cells (2 × 10^5^ cells/well) were seeded in 1 ml of serum-free medium (OPTI-MEM®, Gibco) in a 12-well microtiter plate, and then incubated at 37 °C in a 5% CO_2_ incubator overnight to obtain 80–90% confluence. Cells were pre-washed with serum-free OPTI-MEM® medium and covered with 1 mL of the same medium. Metafectene PRO (Biontex) was complexed with the pEGFP-C1 plasmid at reagent:DNA (v/w) ratio of 4:0.5. Metafectene PRO complex with DNA was added in a volume of 0.1 mL per well and cells were incubated for 48 h at 37 °C in a 5% CO_2_ incubator. Cells were washed three times with sterile PBS; then, 1 mL of fresh PBS was finally added and cells analyzed by confocal microscopy or under UV light in a Nikon microscope (Eclipse E 800). Neuroblastoma SH-SY5Y cells and HepG2 HCC cells were also transfected with plasmids pEGFP-GLS2 and pCMV-Myc-GLS2 by using Lipofectamine 3000 (Thermo Fisher Scientific) at reagent:DNA ratios (v/w) ranging from 1:3 to 2:3, following the manufacturer’s instructions. A pEGFP-24 transfection control plasmid, expressing the GFP protein alone but adding a non-coding spacer nucleotide sequence of 671 pb at the C-terminus of GFP, was also prepared in order to yield a similar molecular mass to the pEGFP-GLS2 plasmid. COS-7 cells were transfected with pEGFP-24 and pEGFP-GLS2 using FUGENE HD (Promega) as transfection reagent. The optimum reagent:DNA ratio (v/w) ratio was 4:0.5. COS-7 cells (5 × 10^4^) were seeded in 24-well plates and transfections performed following the manufacturer’s instructions.

### Preparation of mitochondria and nuclei from human cancer cells

All procedures were carried out at 4 °C or in ice. Nuclear extractions of HepG2 and SH-SY5Y cells were done using a Nuclear and Cytoplasmic Extraction kit (Thermo Scientific) according to the manufacturer’s instructions. To assess mitochondrial contamination, all nuclear fractions were routinely probed by immunoblot analysis with rabbit polyclonal antibody against COX IV (1:3500, Abcam) using cytosolic (mitochondrial) fractions as a positive control. Additionally, α-Tubulin (DM1A) Mouse mAb #3873 (1:1000 dilution, Cell Signaling Technology) was included as a cytoplasmic marker to further assess that nuclear fractions were not contaminated with cytoplasm.

### Immunocytochemistry and confocal microscopy

HepG2, SH-SY5Y and T98G cells (3 × 10^5^ cells) were seeded in six-well culture plates (3 × 10^5^ cells per well) containing a microscope slide in the bottom. COS-7 cells were seeded in 24-well culture plates (5 × 10^4^ cells per well) containing a microscope slide in the bottom. Cells were fixed and immunostained in the exponential phase of growth (60–72 h after initiating the culture). All steps were done at room temperature. The standard protocol for fluorescent labeling of proteins was as follows: microscope slides were rinsed briefly with PBS and then fixed with 4% paraformaldehyde (freshly prepared) in PBS for 20 min. Following fixation, the slides were rinsed twice with PBS and autofluorescence cells blocked with 50 mM NH_4_Cl in PBS for 10 min. In order to remove the remained NH_4_Cl, cells were incubated for 10 min with 20 mM glycine in PBS. Then, cells were permeabilized for 10 min with 0.1% Triton X-100 (v/v) in PBS (0.05% (w/v) sodium deoxycholate in PBS was also included for COS-7 cells). Cells were then blocked with a solution of 4% (w/v) bovine serum albumin (BSA Fraction V) and 10% (v/v) fetal calf serum in PBS (Ab dilution buffer) for 1 h. The slides were then transferred to a solution of the primary antibody in the same buffer and incubated for 1 h. After incubation, the slides were washed 4 × 10 min with PBS and incubated with secondary antibody in Ab dilution buffer for 1 h. After 4 × 10 min washes with PBS, the slides were mounted using PBS/glycerol 1:1 (v/v) with 2% DABCO. Purified anti-GLS2 antibodies were used at a 1:2000 dilution. Experiments with pre-immune serum were always run in parallel. Double fluorescence labeling was carried out as described elsewhere^24,25^, using rabbit polyclonal anti-GLS2 antibodies (see Supplemental Figs. S9 and S10) visualized with Alexa Fluor 488-conjugated goat anti-rabbit IgG (1:1000, Molecular Probes) and DAPI (10 ng/mL) for 5 min to stain nuclei. Affinity-purified rat polyclonal anti-GLS2 antibodies (Fig. S8), which only recognize the cytosolic GLS2, were employed to maximize the mitochondrial GLS2 signal in double immunofluorescence labeling experiments with anti-VAMP8 antibodies (marker of transport vesicles) in HepG2 cells treated with PMA. For the rest of immunocytochemical studies, affinity-purified rabbit polyclonal anti-GLS2 antibodies were always used. As a marker for mitochondria, Alexa Fluor 555-conjugated mouse monoclonal anti-cytochrome c antibody (1:200; clone 6H2/B4, isotype IgG1, BD Biosciences) was used for HepG2 cells, and mouse monoclonal anti-cytochrome c (A-8) antibody (1:100; Santa Cruz Biotechnology) visualized with Alexa 568 anti-mouse (1:1000, Invitrogen) for COS-7 cells. Slides were examined under a Leica-TCS-NT confocal laser microscope using oil immersion 40x objective and pinhole = 1. In all cases, immunolabeling was not observed when the primary antibody was omitted or pre-immune serum was used instead of primary antibody. Furthermore, the immunolabeling with anti-GLS2 antibodies could be completely blocked by pre-incubation with the purified protein antigen (Fig. S8). For subnuclear localization of GLS2, double immunofluorescence labeling was performed with anti-GLS2 antibodies and the following marker antibodies: rabbit polyclonal anti-JMJD5 (C-terminus) for nucleoplasm (Millipore), dilution 1:200; mouse monoclonal anti-SC35 (phospho) antibody [SC-35] for nuclear speckles (Abcam), dilution 1:300; and mouse monoclonal anti-Fibrillarin antibody [38F3] for nucleolus (Abcam), dilution 1:300.

### Cell cycle analysis

SH-SY5Y or T98G cells were seeded in 6-well plates. PMA and DMSO were added 24 hours after seeding and cells collected 12 or 24 h after treatment. In the case of T98G, T98G-pcDNA3 and T98G-GAB cells, they were collected 24 and 48 hours after seeding. After incubation, cells were washed with PBS, trypsinized and collected by centrifugation at 285 g for 10 min. Cell pellets were washed again with 500 µl of PBS-FBS-HEPES solution (1x PBS, 1% FBS, 10 mM HEPES) and centrifuged at 600 g for 5 min. Cells were dispersed in the small volume of PBS and fixed with 1 mL of 70% ice-cold ethanol. The samples were kept at −20 °C overnight. After fixation, cells were centrifuged at 600 g for 10 min and washed with the previous buffer (procedure repeated twice). Finally, fixed cells were resuspended in 600 µL of staining solution (100 µg/mL RNase A and 40 µg/mL propidium iodide in PBS-FBS-HEPES) and incubated in the dark for 30 min at 37 °C. The cell cycle distribution was determined using a BD FACSVerse Flow cytometer (BD Biosciences) and analyzed with Kaluza software (Beckman Coulter Life Sciences).

### Western blotting

Cell extracts and pure GA proteins were analyzed by SDS-PAGE and Western blotting essentially as described^60^. The blots were developed with the Pierce™ SuperSignal West Pico PLUS Chemiluminescent Substrate as recommended by the supplier (Thermo Scientific). The working dilution of the anti-GLS2 antibody was 1:1000. The following commercial antibodies were used: phospho-p53 (Ser15) Antibody: #9284, Cell signaling technology, dilution 1:1000; purified mouse anti-p21: #556430, BD Pharmingen, BD Biosciences, dilution 1:500; anti-c-Myc antibody [Y69]: #ab32072, Abcam, dilution 1:1000; anti-c-Myc tag antibody #ab9106, Abcam, dilution 1:4000; anti-TATA binding protein (TBP) antibody [1TBP18], ChIP Grade (#ab818): Abcam, dilution 1:1000; mouse monoclonal anti-β-actin antibody MAB1501, clone C4, Merck, dilution 1:500.

For Western blotting of brain tissues, a mouse (adult male C57BL/6) whole brain extract was prepared as outlined before^25^. Rat (adult male Sprague-Dawley) prefrontal cortex (PFC) was a kind gift of Dr. Gert Lubec. Whole protein extract of rat PFC was obtained as described elsewhere^62^. All animal experiments were carried out in accordance with the European Union regulations (Council Directive 86/609/ECC of 24 November 1986) and approved by the committee of animal use for research at Malaga University, Spain (RD 1201/2005 of 10 October 2005).

### Heterologous expression of human glutaminases

All the studies with the baculovirus expression system were done with the Sf9 insect cell line derived from *Spodoptera frugiperda*. Recombinant baculovirus for the GLS2 protein was obtained as described before^29^. Aliquots of the virus stock were maintained at −80 °C and 4 °C, following virus titer measurement using the plaque assay technique. For protein production, Sf9 cells were cultured in TNM-FH complete media containing 0.35 g/LNaHCO_3_ and adjusted at pH 6.0. After filter-sterilization, the media was supplemented with 5% (v/v) bovine fetal serum, 100 IU/mL penicillin, 100 μg/mL streptomycin and 0.25 μg/mL amphotericin. Cells were grown in suspension in 250 or 500 mL flasks in a metabolic incubator without CO_2_ exchange at 27 °C. Then, cells were seeded in 140 mm culture plates and infected with the recombinant virus at a multiplicity of infection of 1. The cells were further incubated at 27 °C in TNM-FH insect cell media containing 5% fetal bovine serum before harvesting at 4 days post-infection. The cells were stored at −80 °C until purification. The KGA isoform was also expressed in baculovirus system. The ORF of human KGA isoenzyme was amplified by PCR using the following primers: KGABamHI-forward: 5′-CGCCCGGA**GGATCC**TCCCCTGTTGAG-3′ and KGAHindIII-reverse 5′-**AAGCTTA***ATGATGATGATGATGATG***CTTGTCATCGTCAT**CCAACAATCCATCAAGATTC-3′ (restriction sites in bold, or stop codon underlined). The reverse primer for KGA contained six codons for histidine (indicated in cursive) as well as a recognition site for enteroquinase (shown in bold). The amplified product was cloned into the *Bam*HI and *Hind*III sites of the expression vector pBlueBac4.5 (Invitrogen). Insect cell transfection, recombinant virus isolation and heterologous expression of human KGA-His in Sf9 insect cells was performed as previously described^29^.

A GLS2 protein was designed and expressed in bacteria as negative control for hypusine modification of human GLS2. The deletion construct GLS2_56–602_ was prepared at the NdeI-NotI sites of pET-28b expression vector. The induction of *E. coli* cells was carried out overnight using 1 mM of IPTG at 30 °C. The culture was spun down at 4000 g during 20 min and the pellet was conserved at −80 °C. Also, the recombinant human GIP protein was expressed in bacteria and purified by affinity chromatography as described before^63^.

### Affinity chromatography of human glutaminases

Sf9 cells (150 × 10^6^) were resuspended in 5 mL of buffer I (20 mM Tris, 1 mM EDTA, 250 mM sucrose, pH 8) containing protease inhibitors cocktail (Roche) and supplemented with 1% (v/v) TX-100 for 30 min at 4 °C in a rotary shaker. After centrifuging at 100.000 × g for 10 min the supernatant was applied to the GIP-affinity column. Full details of GIP purification and GLS2 isolation by GIP-affinity chromatography have been previously reported^29^. The affinity resin was packed in small Econo Column (BioRad) and equilibrated with buffer I. After passing the supernatant, the column was washed with 5-column volumes of buffer I and GLS2 was then eluted with a linear NaCl gradient (0–1 M, 10-column volumes) in buffer I. Fractions of 0.5 mL were taken and those showing the highest GA specific activities were pooled and kept at −20 °C until analysis. Recombinant human KGA was purified by immobilized-metal affinity chromatography (IMAC) with a Ni-Sepharose column (Amersham). Infected Sf9 cells (150 × 10^6^ cells) were resuspended in buffer A (20 mM Na_2_HPO_4_ pH 7.4, 500 mM NaCl, 1% (v/v) TX-100, 10 mM imidazol), containing the Roche protease inhibitor cocktail. After incubation for 30 min at 4 °C in a rotary shaker, the extract was centrifuged at 100000 g for 10 min and the supernatant was filtered and passed through a 1 mL Ni-Sepharose column equilibrated in buffer A. The KGA protein was eluted with a linear imidazol gradient (10 mM–500 mM) in buffer A. Fractions were analyzed by SDS-PAGE and enzymatic assay.

Finally, bacterial GLS2_56–602_ was also purified by IMAC chromatography. The pellet was resuspended in lysis buffer (10 mM K_2_HPO_4_, 300 mM KCl, 10 mM imidazole, 10% glycerol, 10 mM Tris-HCl, pH 8, 0.5 mM TCEP) containing protease inhibitor cocktail (Roche) and was sonicated as described elsewhere^60^. Then, the lysed pellets were centrifuged at 20000 g for 1 h, and the soluble extract was clarified using a 0,45-µm syringe filter (ClearLine, Dutscher). The sample was loaded into a HisTrap column (GE Healthcare). Proteins were sequentially washed with 5 column volumes of 75 mM and 100 mM imidazole in buffer 10 mM K_2_HPO_4_, 10 mM Tris-HCl, pH 8, 300 mM KCl, 10% glycerol, 2 mM DTT plus protease inhibitor cocktail. Finally, proteins were eluted with elution buffer (20 mM Na_2_HPO_4_, 500 mM NaCl, 450 mM imidazole, 10% glycerol, 2 mM DTT).

### In-solution proteolytic digestion

Two micrograms of the purified GLS2 protein sample were digested with trypsin or chymotrypsin (1:100 w/w) using the filter-aided sample preparation (FASP) as previously described with minor modifications^64,65^. All digests were desalted and concentrated with customized reversed-phase C18 stage tips^66^. Lyophilized peptides were reconstituted in 5% formic acid and analyzed by LC-MS/MS.

### LC–MS/MS analysis using Orbitrap Q-Exactive HF

Mass spectrometry was performed on a hybrid linear trap quadrupole Orbitrap Q-Exactive HF spectrometer (ThermoFisher Scientific, Waltham, MA) using the Xcalibur version 2.1.0 coupled to an Agilent 1200 HPLC nanoflow system via a nanoelectrospray ion source using liquid junction (Proxeon, Odense, Denmark). Solvents for liquid chromatography-mass spectrometry separation of the digested samples were as follows: solvent A consisted of 0.4% formic acid in water and solvent B consisted of 0.4% formic acid in 70% methanol and 20% isopropanol. From a thermostatic micro-autosampler, 8 μL of the tryptic peptide mixture were automatically loaded onto a trap column (Zorbax 300SB-C18 5 μm, 5 × 0.3 mm, Agilent) with a binary pump at a flow rate of 45 μL/min. 0.1% TFA was used for loading and washing the precolumn. After washing, the peptides were eluted by back-flushing onto a 16 cm fused silica analytical column with an inner diameter of 50 μm packed with C18 reversed phase material (ReproSil-Pur 120 C18-AQ, 3 μm, Dr. Maisch, Ammerbuch-Entringen, Germany). The peptides were eluted from the analytical column with a 27 min gradient ranging from 3 to 30% solvent B, followed by a 25 min gradient from 30 to 70% solvent B, and, finally, a 7 min gradient from 70 to 100% solvent B at a constant flow rate of 100 nL/min. The analyses were performed in a data-dependent acquisition mode using a top 15 CID or HCD method. Dynamic exclusion for selected ions was 60 s. A single lock mass at m/z 445.120024 was employed. The maximal ion accumulation time for MS in the Orbitrap and MS2 in the linear trap was 500 and 50 ms, respectively. Automatic gain control was used to prevent overfilling of the ion traps. For MS and MS2, automatic gain control was set to 10^6^ and 5,000 ions, respectively. Peptides were detected in MS mode at a resolution of 60000 (at m/z 400). The threshold for switching from MS to MS2 was 2,000 counts. All samples were analyzed as technical, back-to-back replicates.

Raw data were interpreted using Proteome Discoverer 2.1 (Thermo Scientific) and proteins were identified using the Mascot search engine (Matrix Science, London, UK). Spectra were matched against the SwissProt/UniProt human (*Homo sapiens*) protein database. The MS/MS ion search parameters were the following: the number of maximum missed cleavages for trypsin was 2; peptide mass tolerance was ±10 ppm (#^13^C = 1) and fragment mass tolerance was ±0.1 Da; carbamidomethylation of cysteine residues and oxidation of methionine residues were determined as fixed and variable modifications, respectively. Additional search using hypusine, deoxyhypusine and acetylhypusine modifications on lysine residues was performed. The significant threshold for matched peptides was given as p < 0.05. Protein identity was considered as verified if it was present at least with two unique peptide sequences.

### Statistical analysis

Statistical analysis was performed with GraphPad Prism5 software. Unless indicated otherwise, data are expressed as the mean ± standard deviation (SD) of three independent experiments. Experimental data were analyzed using one-way analysis of variance (ANOVA) followed by the Tukey’s test. For single comparison between two groups, paired Student’s *t* test was also used.

## Supplementary information

SUPPLEMENTAL FIGURES 1–10, LEGENDS AND SUPPLEMENTAL TABLE 1.

Supplementary TABLE 2.

## Data Availability

The datasets used and/or analysed during the current study are available from the corresponding author on reasonable request. All data generated or analysed during this study are included in this published article [and its supplementary information files].
