# Author Correction: Nuclear Translocation of Glutaminase GLS2 in Human Cancer Cells Associates with Proliferation Arrest and Differentiation

**DOI:** 10.1038/s41598-020-80651-0

**Published:** 2021-01-04

**Authors:** Amada R. López de la Oliva, José A. Campos-Sandoval, María C. Gómez-García, Carolina Cardona, Mercedes Martín-Rufián, Fernando J. Sialana, Laura Castilla, Narkhyun Bae, Carolina Lobo, Ana Peñalver, Marina García-Frutos, David Carro, Victoria Enrique, José C. Paz, Raghavendra G. Mirmira, Antonia Gutiérrez, Francisco J. Alonso, Juan A. Segura, José M. Matés, Gert Lubec, Javier Márquez

**Affiliations:** 1 Departamento de Biología Molecular Y Bioquímica, Canceromics Lab, Facultad de Ciencias, Universidad de Málaga, 29071 Málaga, Spain and Instituto de Investigación Biomédica de Málaga (IBIMA), Málaga, Spain; 2 Proteomics Lab, Central Facility Core, University of Málaga, 29071 Málaga, Spain; 3 Private Medical University of Salzburg, A5020 Salzburg, Austria; 4 Institute of Science and Technology Austria, Am Campus 1, A-3400 Klosterneuburg, Austria; 5 Department of Pediatrics, Indiana University School of Medicine, Indianapolis, IN 46202 USA; 6 Departamento de Biología Celular, Genética Y Fisiología, Facultad de Ciencias, Universidad de Málaga, Instituto de Investigación Biomédica de Málaga (IBIMA). Centro de Investigación Biomédica en Red sobre Enfermedades Neurodegenerativas (CIBERNED), 29071 Málaga, Spain

Correction to: *Scientific Reports* 10.1038/s41598-020-58264-4, published online 10 February 2020

This Article contains errors.

In the Results section under subheading ‘Posttranslational modification of human GLS2 protein’,

“With regard to posttranslational modifications, LC-MS/MS analysis identified the chymotryptic peptide – KEKKCFPKGVDMMAAL - being modified with acetylhypusine in the fourth lysine residue (K-336 of the whole GLS2 amino acid sequence) (Fig. 6-A).”

should read:

“With regard to posttranslational modifications, LC-MS/MS analysis identified the chymotryptic peptide – KEKKCFPKGVDMMAAL - being modified with acetylhypusine in the fourth amino acid residue (K-332 of the whole GLS2 amino acid sequence) (Fig. 6-A).”

In the last paragraph of the Discussion section,

“The hypusine-modified lysine in the GAB primary structure (K-336) is located in an unfolded segment of the protein, a short turn of 9 residues connecting two alpha helices in a sequence highly enriched in polar amino acids (KKCFPKGVD), which strongly suggest that this hypusinated lysine is exposed to the external medium.”

should read:

"The hypusine-modified lysine in the GAB primary structure (K-332) is located in an unfolded segment of the protein, a short turn of 9 residues connecting two alpha helices in a sequence highly enriched in polar amino acids (KKCFPKGVD), which strongly suggest that this hypusinated lysine is exposed to the external medium.”

In the Methods section the subheading ‘LC-MS/MS analysis using Orbitrap Q-Exactive HF’

should read:

‘LC-MS/MS analysis using a Thermo Orbitrap Velos’

Under the same subheading,

“Mass spectrometry was performed on a hybrid linear trap quadrupole Orbitrap Q-Exactive HF spectrometer (ThermoFisher Scientific, Waltham, MA) using the Xcalibur version 2.1.0 coupled to an Agilent 1200 HPLC nanoflow system via a nanoelectrospray ion source using liquid junction (Proxeon, Odense, Denmark).”

should read:

“Mass spectrometry was performed on a Thermo LTQ-Orbitrap Velos spectrometer (ThermoFisher Scientific, Waltham, MA) using the Xcalibur version 2.1.0 coupled to an Agilent 1200 HPLC nanoflow system via a nanoelectrospray ion source using liquid junction (Proxeon, Odense, Denmark).”

Finally, the Data Availability statement is incomplete.

“The datasets used and/or analysed during the current study are available from the corresponding author on reasonable request. All data generated or analysed during this study are included in this published article [and its supplementary information files].”

should read:

“The mass spectrometry proteomics data have been deposited to the ProteomeXchange Consortium via the PRIDE partner repository with the dataset identifier PXD021033. The datasets used and/or analysed during the current study are available from the corresponding author on reasonable request. All data generated or analysed during this study are included in this published article [and its supplementary information files].”

