# Supplementary material for: Nuclear Translocation of Glutaminase GLS2 in Human Cancer Cells Associates with Proliferation Arrest and Differentiation
Source: Sci Rep. 2020 Feb 10;10:2259. doi: 10.1038/s41598-020-58264-4 (PMC7010782; doi:10.1038/s41598-020-58264-4)
Supplement: Supplementary file 1 — SUPPLEMENTAL FIGURES 1–10, LEGENDS AND SUPPLEMENTAL TABLE 1. [file 41598_2020_58264_MOESM1_ESM.pdf]

NUCLEAR TRANSLOCATION OF GLUTAMINASE GLS2 IN HUMAN CANCER CELLS ASSOCIATES WITH PROLIFERATION ARREST AND DIFFERENTIATION

Amada R. López de la Oliva[#‡], José A. Campos-Sandoval[§‡], María C. Gómez-García[#‡], Carolina Cardona[#], Mercedes Martín-Rufián[§], Fernando J. Sialana[Ω], Laura Castilla[#], Narkhyun Bae[π], Carolina Lobo[§], Ana Peñalver[#], Marina García-Frutos[#], David Carro[#], Victoria Enrique[#], José C. Paz[#], Raghavendra G. Mirmira[€], Antonia Gutiérrez[‖], Francisco J. Alonso[#], Juan A. Segura[#], José M. Matés[#], Gert Lubec[Ω] and Javier Márquez[#]*

FIGURE S1

Figure 1 D

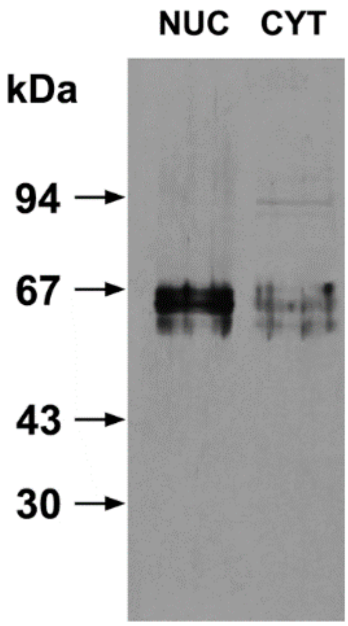

Figure 2 C

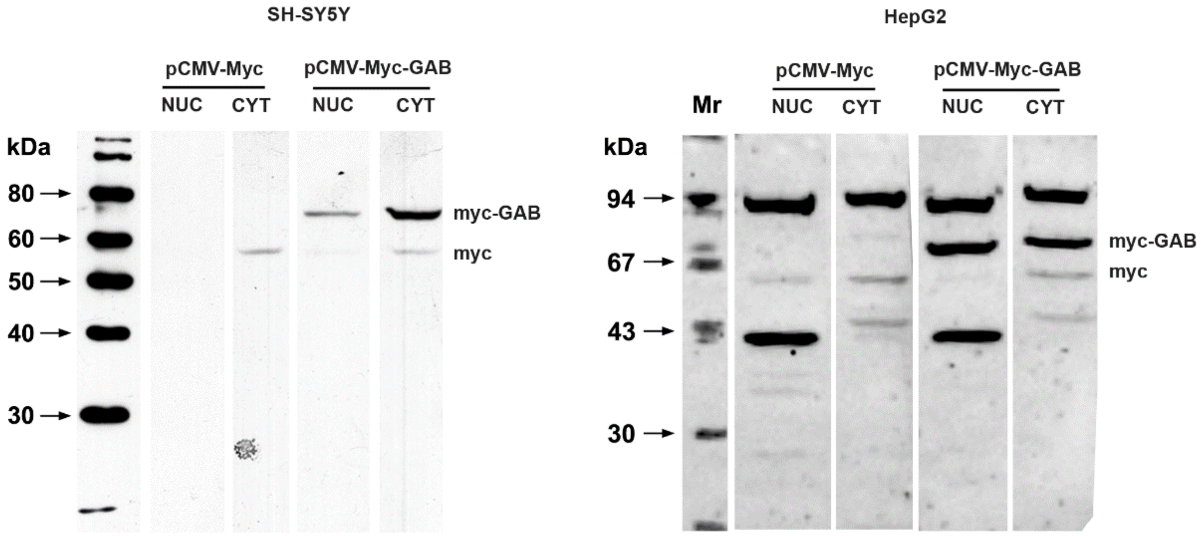

FIGURE S1

Figure 2 D

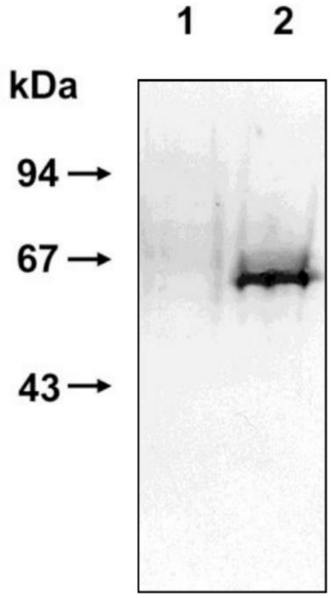

Figure 3 A

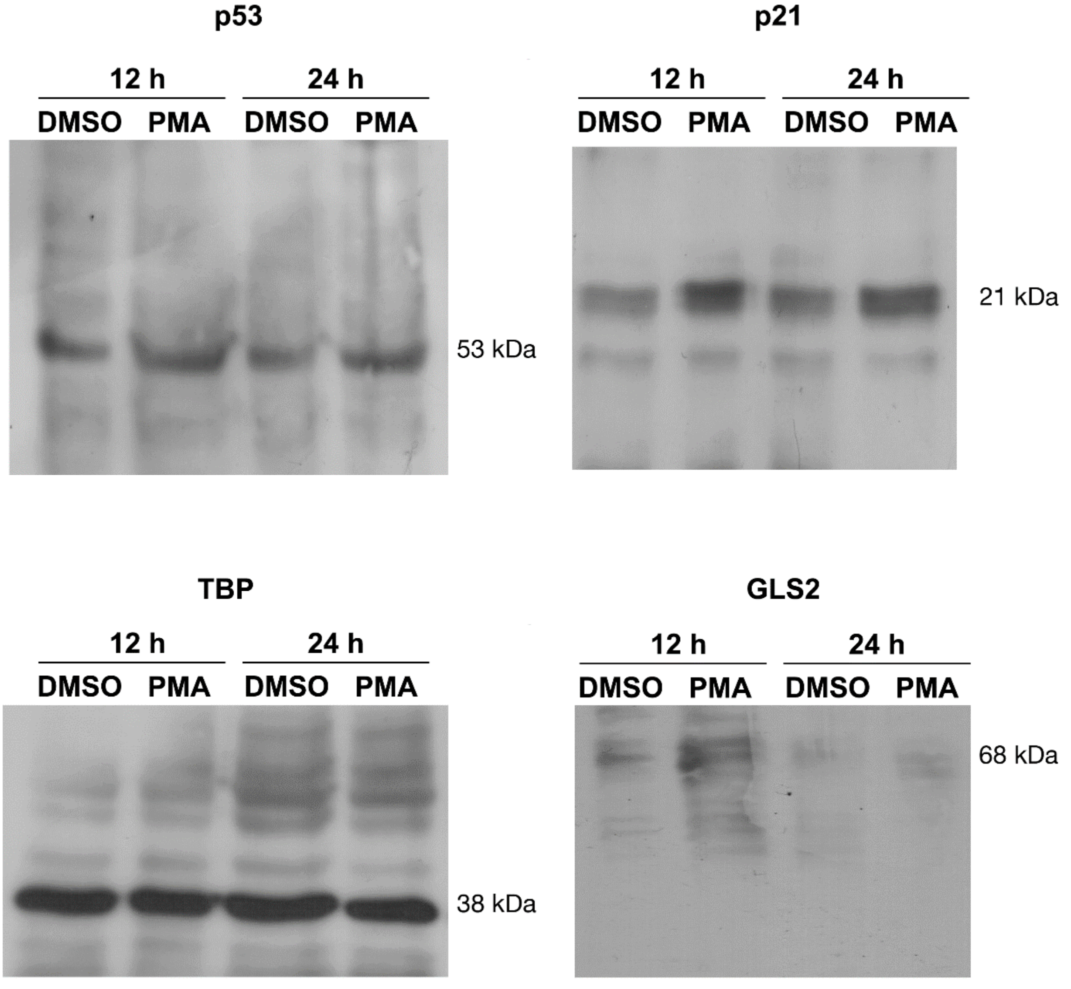

FIGURE S1

Figure 3 B

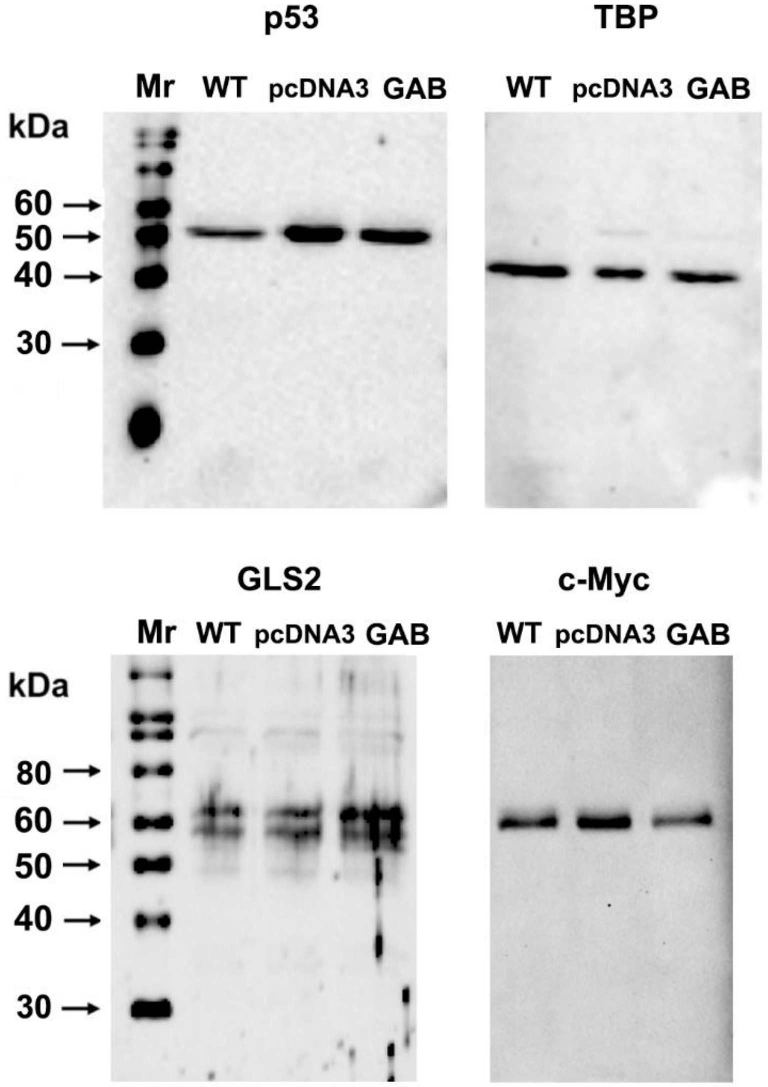

Figure 3 C

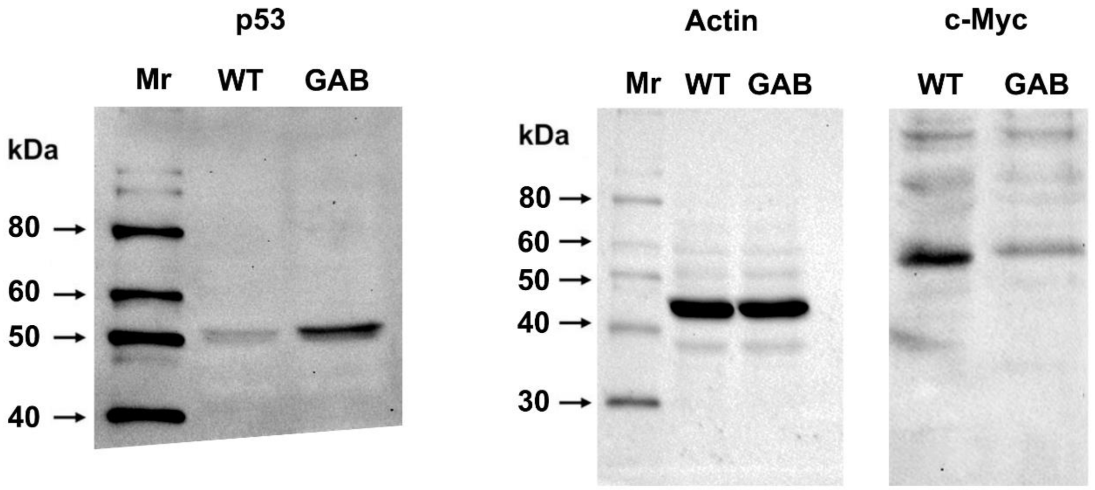

FIGURE S1

1. From Figure 1 (bottom)

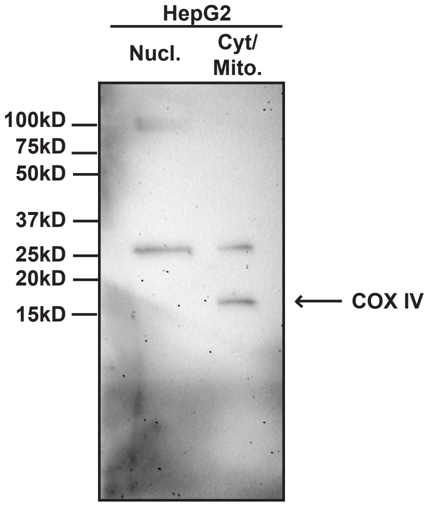

2. From Figure 2C

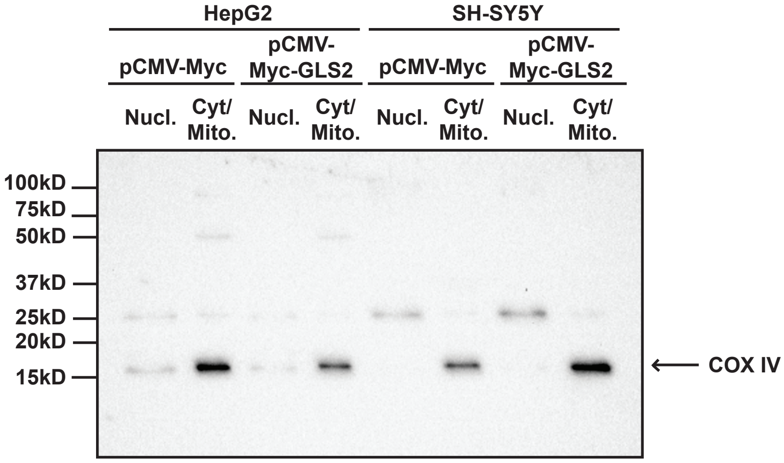

3. From Figure 2D

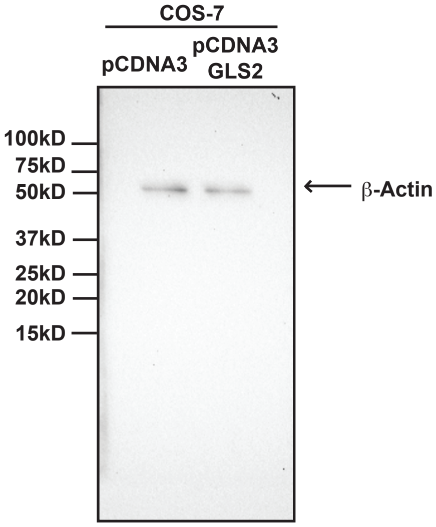

FIGURE S2

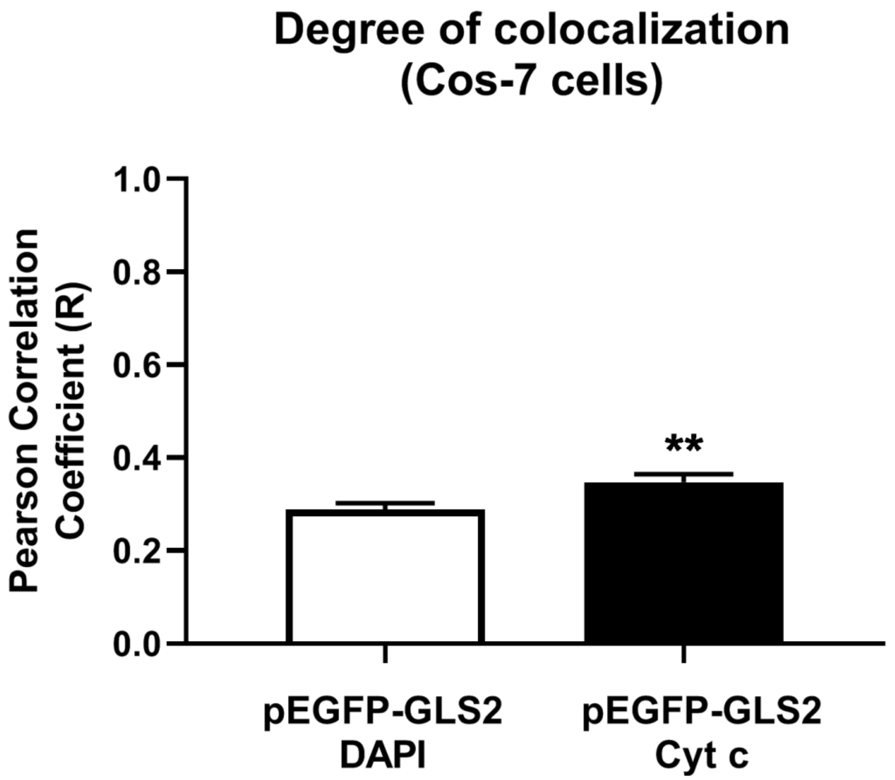

Degree of colocalization
(Cos-7 cells)
Pearson Correlation Coefficient (R)
1.0
0.8
0.6
0.4
0.2
0.0
**
pEGFP-GLS2
DAPI
pEGFP-GLS2
Cyt c

FIGURE S3

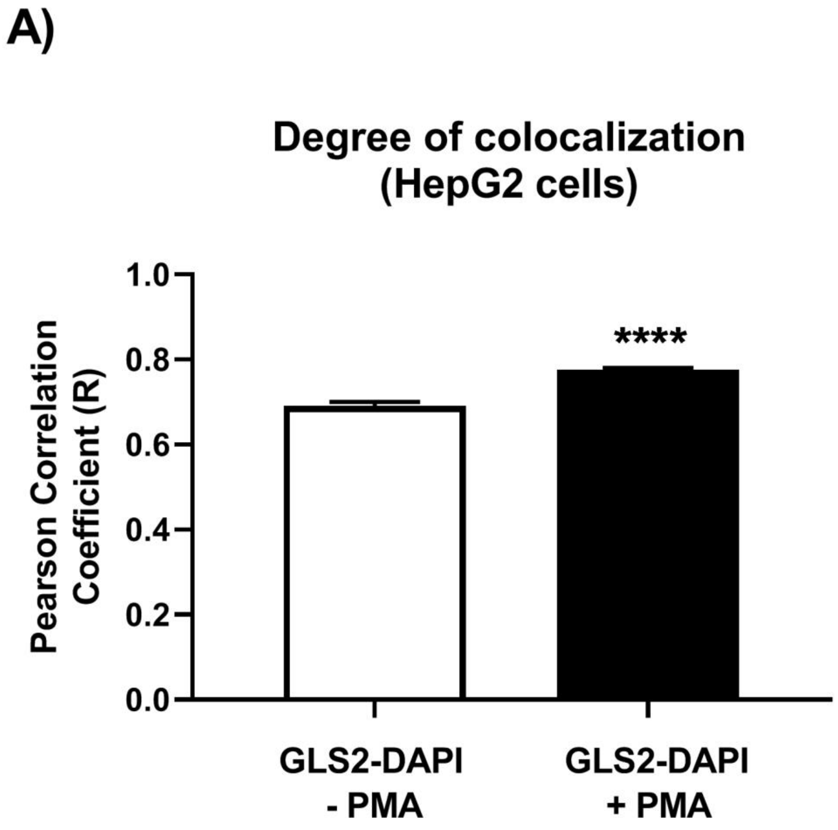
A)
Degree of colocalization
(HepG2 cells)
Pearson Correlation
Coefficient (R)
1.0
0.8
0.6
0.4
0.2
0.0
****
GLS2-DAPI
- PMA
GLS2-DAPI
+ PMA

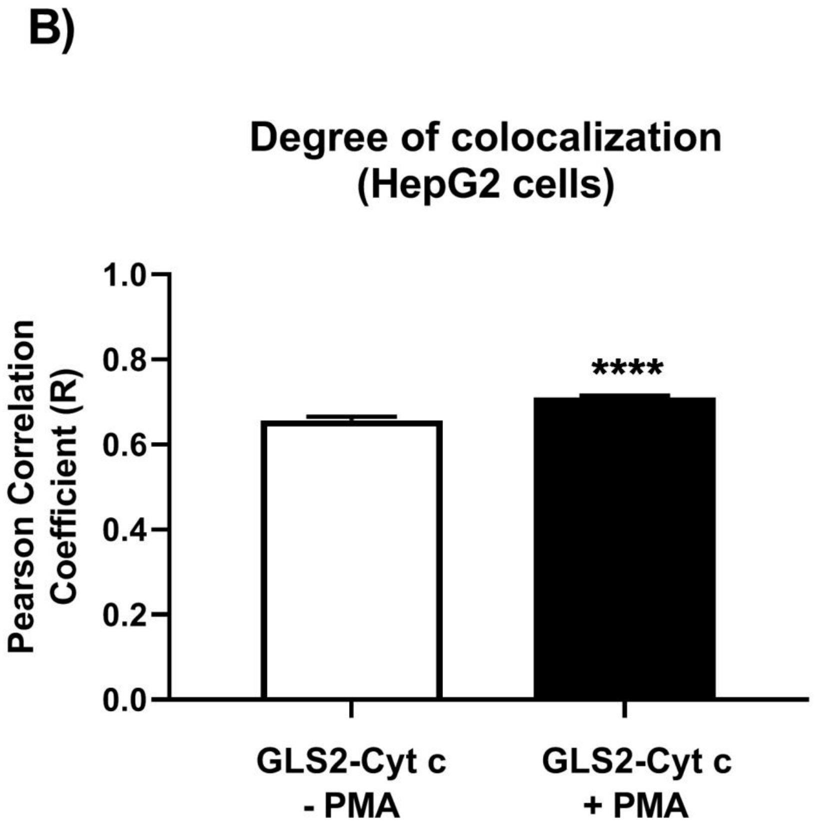
B)
Degree of colocalization
(HepG2 cells)
Pearson Correlation
Coefficient (R)
1.0
0.8
0.6
0.4
0.2
0.0
****
GLS2-Cyt c
- PMA
GLS2-Cyt c
+ PMA

FIGURE S4

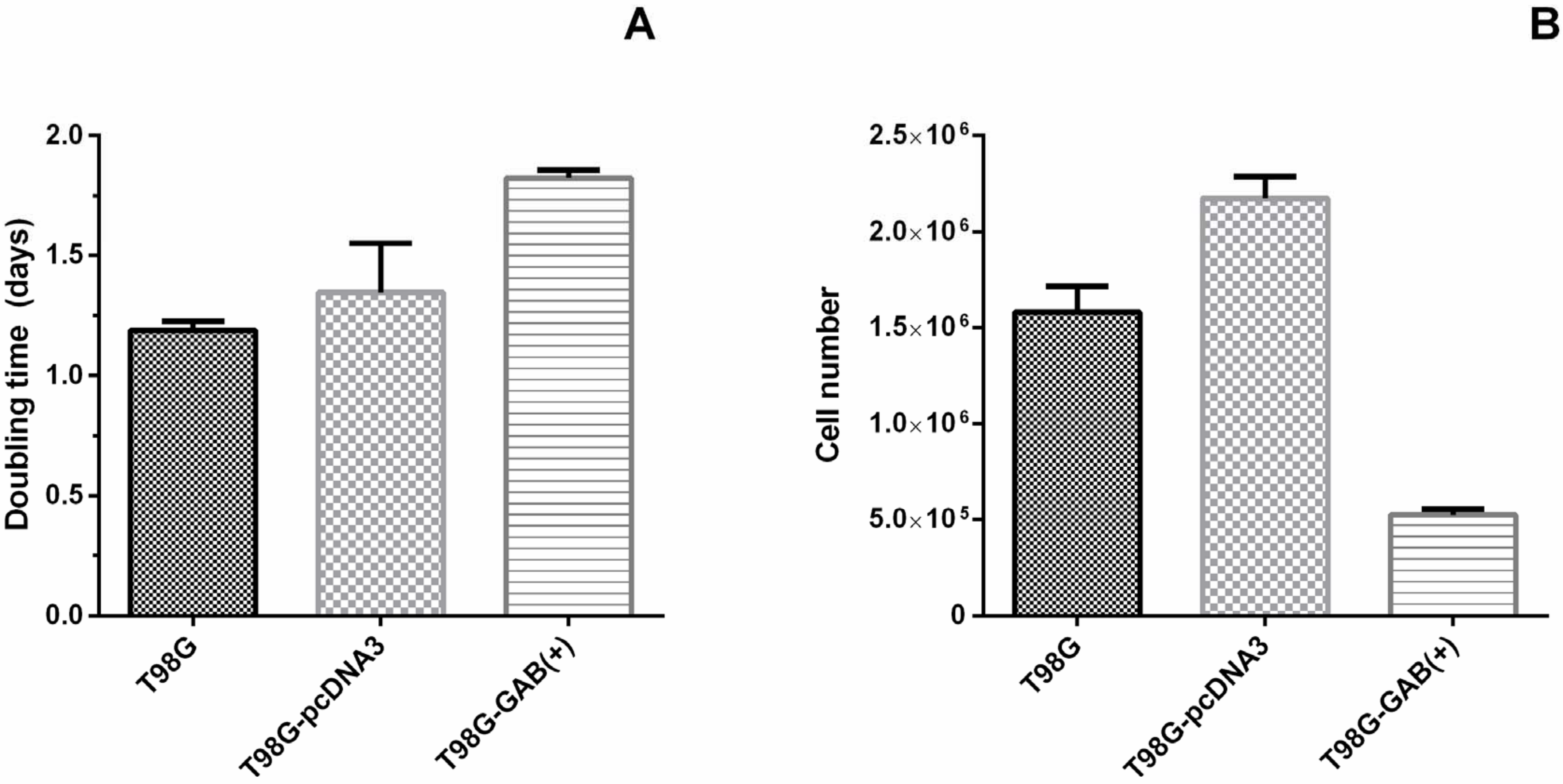
A
Doubling time (days)
2.0
1.5
1.0
0.5
0.0
T98G
T98G-pcDNA3
T98G-GAB(+)
B
Cell number
2.5×10^6
2.0×10^6
1.5×10^6
1.0×10^6
5.0×10^5
0
T98G
T98G-pcDNA3
T98G-GAB(+)

FIGURE S5

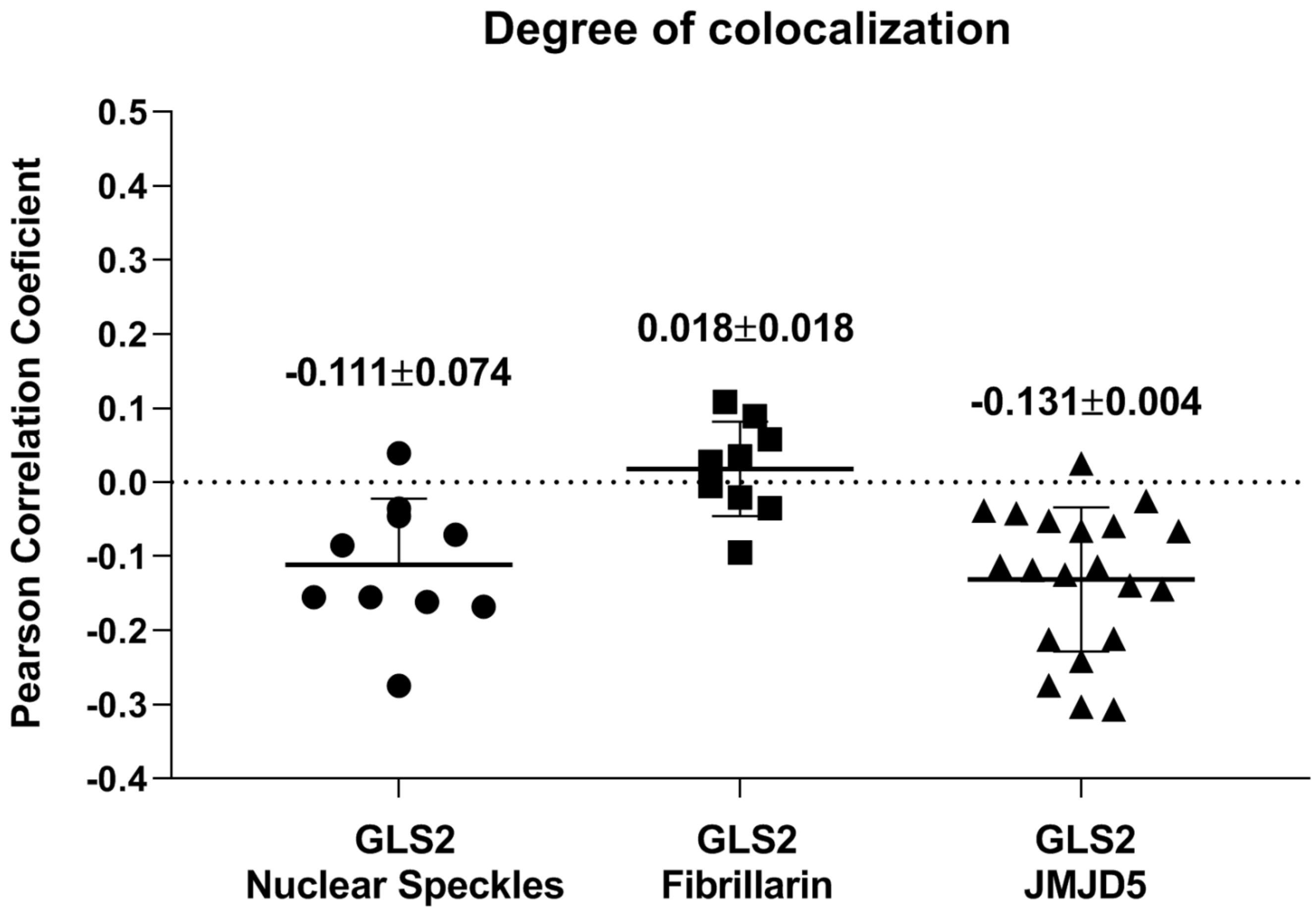
Degree of colocalization
Pearson Correlation Coeficient
0.5
0.4
0.3
0.2
0.1
0.0
-0.1
-0.2
-0.3
-0.4
-0.111±0.074
0.018±0.018
-0.131±0.004
GLS2
Nuclear Speckles
GLS2
Fibrillarin
GLS2
JMJD5

FIGURE S6

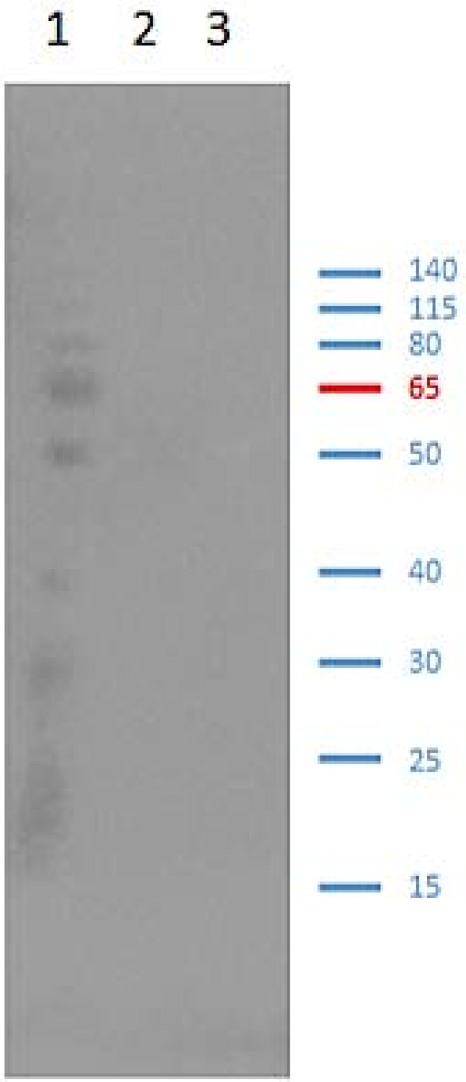

FIGURE S7

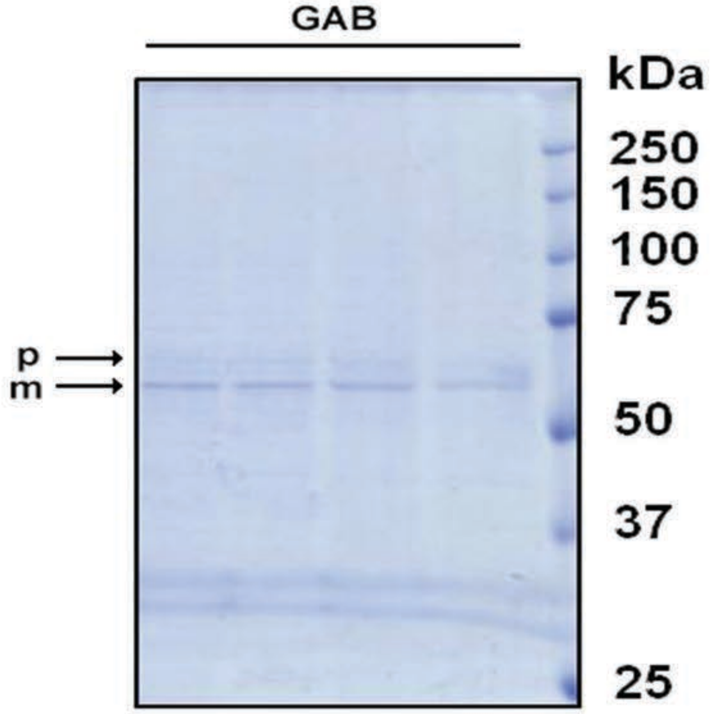

FIGURE S8

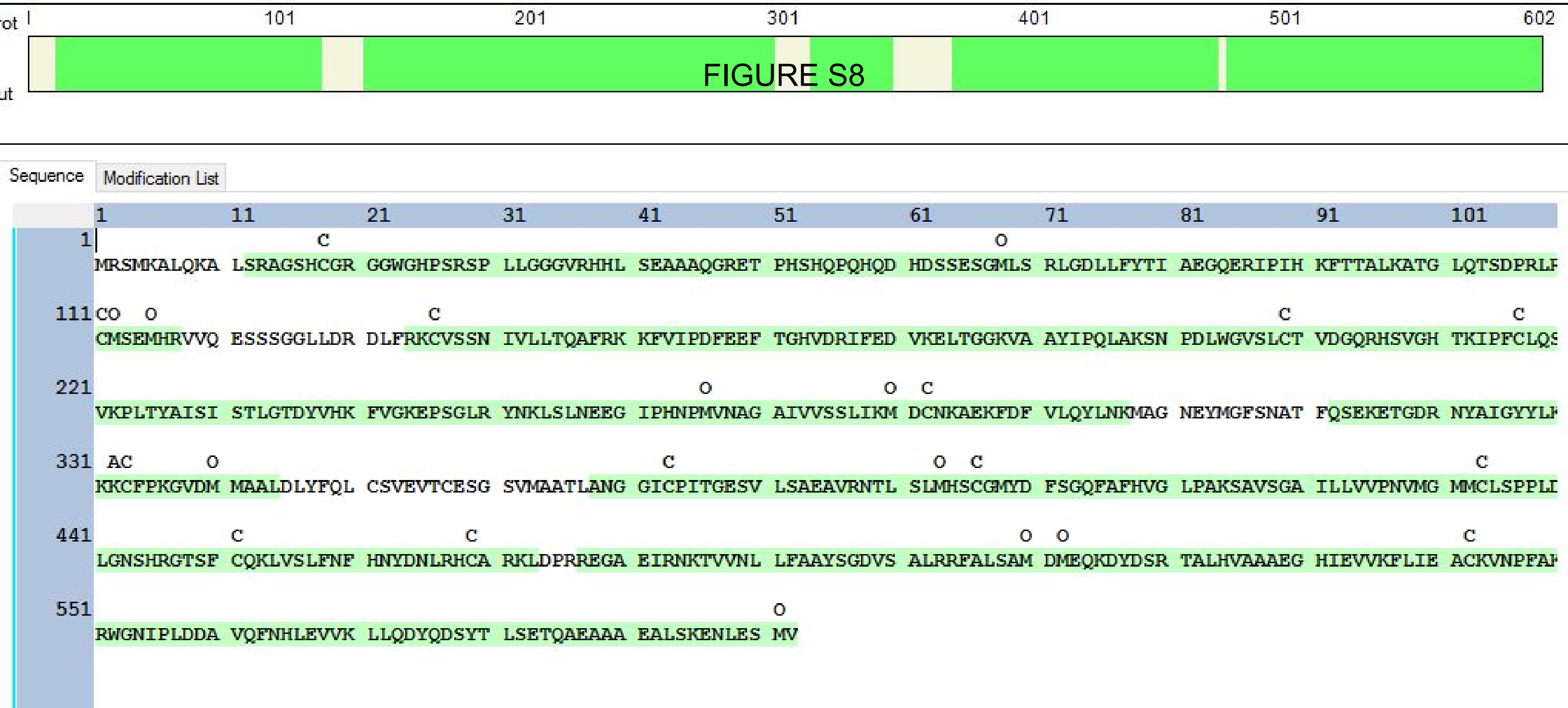

1
101
201
301
401
501
602
Sequence
Modification List
1 11 21 31 41 51 61 71 81 91 101
1
C O
MRSMKALQKA LSRAGSHCGR GGWGHPSRSP LLGGGVRHHL SEAAAQGRET PHSHQPQHQD HDSSESGMLS RLGDLLFYTI AEGQERIPIH KFTTALKATG LQTSDPRLF
111
CO O C C C
CMSEMHRVVQ ESSSGGLLDR DLFRKCVSSN IVLLTQAFRK KFVIPDFEEF TGHVDRIFED VKELTGGKVA AYIPQLAKSN PDLWGVSLCT VDGQRHSVGH TKIPFCLQS
221
O O C
VKPLTYAISI STLGTDYVHK FVGKEPSGLR YNKLSLNEEG IPHNPMVNAG AIVVSSLIKM DCNKAEKFDF VLQYLNKMAG NEYMGFSNAT FQSEKETGDR NYAIGYYLF
331
AC O C O C C
KKCFPKGVDM MAALDLYFQL CSVEVTCESG SVMAATLANG GICPITGESV LSAEAVRNTL SLMHSCGMYD FSGQFAFHVG LPAKSAVSGA ILLVVPNVMG MMCLSPPLI
441
C C O O C
LGNSHRGTSF CQKLVSLFNF HNYDNLRHCA RKLDPRREGA EIRNKTVVNL LFAAYSGDVS ALRRFALSAM DMEQKDYDSR TALHVAAAEG HIEVVKFLIE ACKVNPFAK
551
O
RWGNIPLDDA VQFNHLEVVK LLQDYQDSYT LSETQAEAAA EALSKENLES MV

FIGURE S9

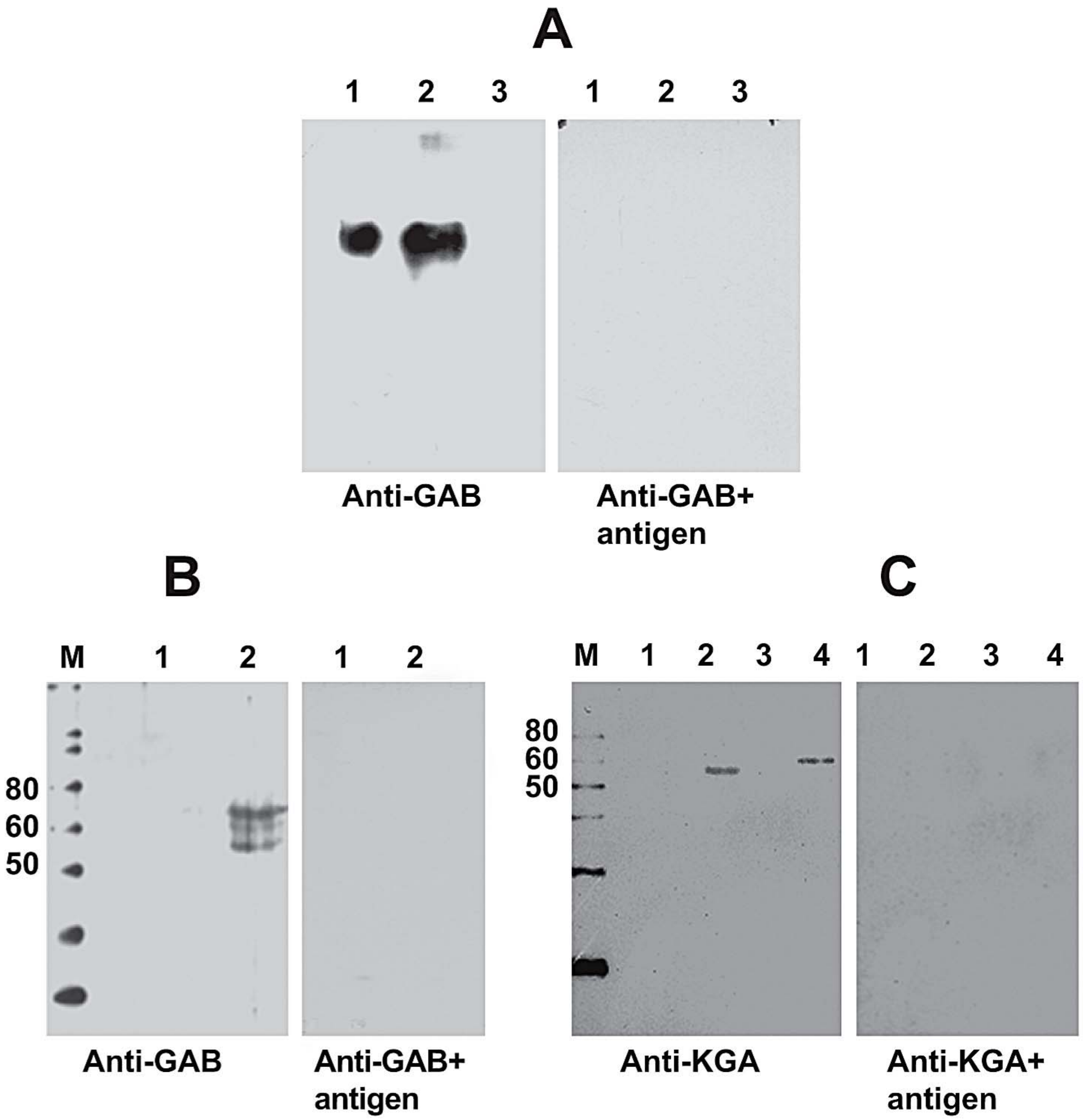
A
1 2 3 1 2 3
Anti-GAB
Anti-GAB+ antigen
B
M 1 2 1 2
80
60
50
Anti-GAB
Anti-GAB+ antigen
C
M 1 2 3 4 1 2 3 4
80
60
50
Anti-KGA
Anti-KGA+ antigen

FIGURE S10

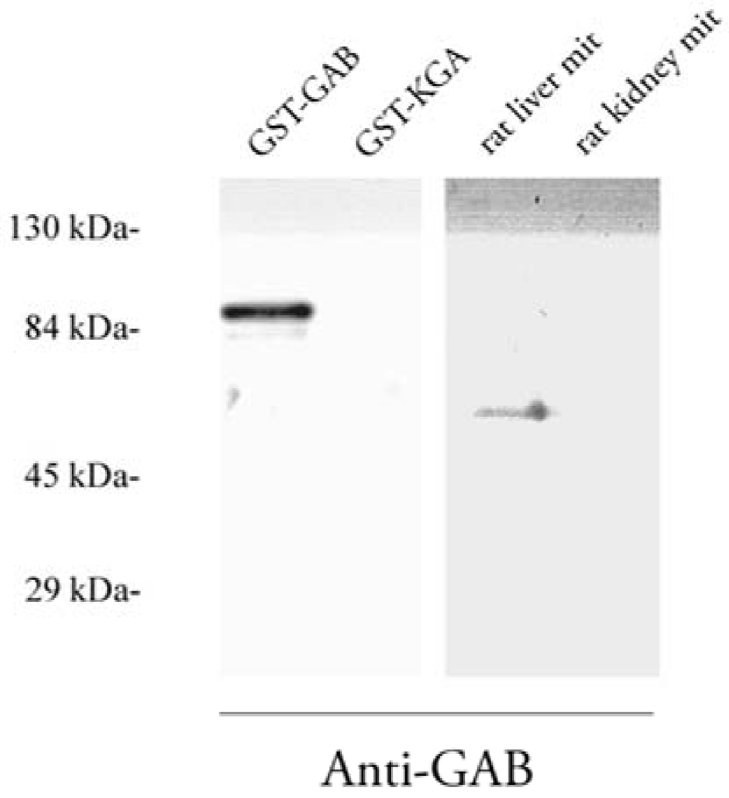
GST-GAB
GST-KGA
rat liver mit
rat kidney mit
130 kDa-
84 kDa-
45 kDa-
29 kDa-
Anti-GAB

SUPPLEMENTAL FIGURE 1. Uncropped full-length Western blots shown in the Results section. In addition to the key protein targets assayed by immunoblot analyses, samples were also probed for loading control (β-actin) and for markers of different subcellular fractions: tubulin for cytosol, TATA-binding protein for nucleus and cytochrome c oxidase for mitochondria.

SUPPLEMENTAL FIGURE 2. Fluorescence co-localization analysis of EGFP-GLS2 in COS-7 cell nuclei and mitochondria. Degree of co-localization of EGFP-GLS2 protein in mitochondria and nuclei of COS-7 cells was quantified using IMAGE J Fiji software. The Pearson correlation coefficients (R) of pixel intensities of GLS2/DAPI and GLS2/cyt c stainings were 0.29 and 0.35, respectively. The regions of interest (ROI) analyzed were the nucleus and the whole cell, respectively (n = 120 individual cells per condition). **p <0.01, unpaired t test.

SUPPLEMENTAL FIGURE 3. Fluorescence co-localization analysis of GLS2 in HepG2 cell nuclei and mitochondria: effect of PMA. Degree of co-localization of GLS2 protein in mitochondria and nuclei of HepG2 cells was quantified using IMAGE J Fiji software. The Pearson correlation coefficients (R) of pixel intensities for GLS2/DAPI in control and PMA-treated cells were 0.69 and 0.77, respectively. For the GLS2/cyt c immunofluorescence stainings, Pearson correlation values of 0.65 and 0.71 were obtained in the absence or presence of PMA, respectively. The regions of interest (ROI) analyzed were the nucleus and the whole cell, respectively (n ≥ 75 individual cells per condition). ****p <0.0001, unpaired t test.

SUPPLEMENTAL FIGURE 4. Growth and proliferation characteristics of T98G cell lines. A. For doubling time calculation, three different curves were represented for each cell line variant (wild-type T98G, sham-transfected T98G-pcDNA3 and GAB-transfected T98G-GAB) by randomly choosing one of the three different cell counting measured for each day. Doubling time per day was calculated for each of these curves and the mean value and standard deviation obtained from these three calculations is shown in a column diagram for each cell line. Mean doubling times were: 1,188 ± 0,039 (T98G), 1,345 ± 0,207 (T98G-pcDNA3) and 1,824 ± 0,034 (T98G-GAB). **B**. To assess the behavior of T98G cancer cell model against nutrient stress, the three glioma T98G cell lines were also cultured in adult bovine serum for 7 days, as described in the Methods section. A representative experiment is shown done by triplicate. Bars indicate the total number of cells (5 x 10^5 cells seeded) after 7 days of culture.

SUPPLEMENTAL FIGURE 5. Fluorescence co-localization analysis of GLS2 in subnuclear organelles of HepG2 cells. Degree of co-localization of GLS2 protein in nuclear speckles, nucleolus and nucleoplasm of HepG2 cells was quantified with IMAGE J Fiji software. The Pearson correlation coefficients (R) of pixel intensities of GLS2/nuclear speckles, GLS2/fibrillarin and GLS2/JMJD5 stainings were -0.11, 0.02 and -0.13, respectively. The region of interest (ROI) analyzed was the nucleus (n= 10-20 individual cells per condition).

SUPPLEMENTAL FIGURE 6. Immunoblot analysis of protein hypusination: additional negative controls. Purified human recombinant GIP protein expressed in bacteria and human recombinant KGA protein expressed in baculovirus were purified by affinity chromatography, as detailed in the Methods section, and used as additional non-hypusinated negative controls. In parallel with the Western blot shown in Fig. 6, we analyzed this membrane containing GIP and KGA-His (20 µg of each protein) which was revealed with anti-hypusine antibodies (1:1000 dilution). A 12% polyacrylamide Bis-Tris gel and PageRuler Prestained Protein Ladders (Thermo Scientific) were used (marker positions indicated at the right end). Lane 1: molecular mass markers; lane 2: GIP protein; lane 3: KGA-His protein.

SUPPLEMENTAL FIGURE 7. SDS-PAGE of purified GLS2 protein fractions submitted to mass spectrometry and immunoblot analyses for detection of posttranslational modifications. The human GAB glutaminase isoform encoded by the *GLS2* gene was expressed in baculovirus system as described in Materials and Methods and reference #29. Precursor (p) and mature (m) forms of recombinant human GAB were purified after GIP-affinity purification, with the mature protein being predominant (reference #29). Purified fractions were analyzed by SDS-PAGE gel and stained with Coomassie blue. Standard molecular weight markers are shown at the right.

SUPPLEMENTAL FIGURE 8. Sequence coverage for GLS2 pure protein fractions analyzed by nano-HPLC-Orbitrap. The amino acid sequence of the GLS2 protein (SwissProt #Q9UI32, 602 amino acids) is shown on top. The number of amino acids that were found in the peptides sequenced in our MS/MS study are shown in green and account for 89% of total sequence coverage.

SUPPLEMENTAL FIGURE 9. Isoform-specific anti-GA antibodies. The specificity and reactivity of rabbit polyclonal anti-KGA and anti-GAB antibodies, and rat polyclonal anti-GAB antibodies, used throughout this study were proved by Western blot. The indicated human recombinant KGA and GAB proteins (10 ng), total extracts from rat liver and brain (35-40 µg) as well as rat liver and rat kidney mitochondria (10-20 µg) were subjected to SDS-PAGE and immunoblotting as described under "Materials and Methods" and probed with anti-GA antibodies. **A**: Rabbit polyclonal anti-GAB (dilution 1:2000); left panel, lane 1, recombinant GAB; lane 2, rat liver extract; lane 3, recombinant KGA; right panel, similar blot revealed with antibody that had been preadsorbed with the indicated antigen. **B**: Rat polyclonal anti-GAB (dilution 1:2000); left panel, lane M, molecular mass markers; lane 1, recombinant KGA; lane 2, rat liver mitochondria; right panel, similar blot revealed with antibody that had been preadsorbed with the indicated antigen. **C**: Rabbit polyclonal anti-KGA (1:1000 dilution); left panel, lane M, molecular mass markers; lane 1, recombinant GAB; lane 2, rat brain extract; lane 3, rat liver extract; lane 4, rat kidney mitochondria. The right panels in A, B and C show similar blots probed with antibody that had been preadsorbed with the indicated antigen.

SUPPLEMENTAL FIGURE 10. Isoform-specific anti-GAB antibodies. Affinity-purified abbit polyclonal anti-GAB antibodies were probed against the indicated GST fusion proteins (100 ng) as well as rat liver and rat kidney mitochondria (20 µg). Proteins were analyzed by SDS-PAGE and immunoblotting as described under "Materials and Methods".

Supplementary Table 1. LC-MS analyses of purified recombinant human GLS2 protein with different MS/MS fragmentation and protease digestion.

**Protease**	**MS/MS Fragmentation**	**Sequence Coverage**	**# Unique Peptides**	**# Peptides**	**# PSMs**
Trypsin	CID	66.1	31	31	130
Trypsin	HCD	77.1	52	54	1102
Chymotrypsin	CID	57.6	45	45	119
Chymotrypsin	HCD	72.6	69	69	838
TOTAL		88.9	122	126	3299
